# Spirometry practice and the impact of a phase 1 training workshop among health workers in southern Nigeria: a cross-sectional study

**DOI:** 10.1186/s12890-020-01291-8

**Published:** 2020-10-06

**Authors:** Adaeze Ayuk, Chizalu Ndukwu, Samuel Uwaezuoke, Eno Ekop

**Affiliations:** 1 College of Medicine, University of Nigeria Ituku-Ozalla Enugu Campus, Enugu, Nigeria; 2 Department of Pediatrics, University of Nigeria Teaching Hospital, Ituku-Ozalla, Enugu, Nigeria; 3 Department of Paediatrics, College of Medicine, Nnamdi Azikiwe University, Awka, Anambra Nigeria; 4 College of Health Science, University of Abuja, Abjua, Nigeria; 5 Department of Paediatrics, Gwagwalada Teaching Hospital Abuja FCT, Abuja, Nigeria

**Keywords:** Competency, Practice, Spirometry, Training, Workshop, Nigeria

## Abstract

**Background:**

Global standards require that spirometry should be performed by trained and experienced personnel, who would be able to assess the correct performance of tests by patients and assure good quality of the result. The complete achievement of this requires a two-step assessment where competency in both knowledge and skills are tested. This study aims to assess the impact of a one-day hands-on spirometry training (Phase1), on the knowledge and application of spirometry among health workers.

**Methods:**

This was a descriptive cross-sectional study, which describes a one-day (seven hours) spirometry training and skills impartation done at two conference city locations in Enugu and Calabar in the southern part of Nigeria. All the verbally consenting attendees who completed the training assessment tests constituted the study population. The assessment of the spirometry knowledge base before and after the theory and practical sessions, on the various aspects of spirometry, according to international best practices, quality assurance and the interpretation of results, was done and the outcome was analyzed. Factors that could affect the outcome were also assessed.

**Results:**

There were 64 consenting participants of whom 54.7% (35/64) were females. Theparticipants demonstrated much improved post-intervention knowledge and could satisfactorily perform spirometry, calibration, interpretation of test results and quality control as evidenced by the post test scores after practical sessions were conducted. Pre-test mean scores improved by a mean difference of 12% (*p* < 001) and were affected by the year of academic graduation and availability of spirometers at the place of work, an effect that was no longer seen at post-test following the hands-on spirometry training.

**Conclusion:**

The present study has shown that a one-day spirometry workshop significantly improved the knowledge of spirometry practice. There is need to set up more frequent locally-organized spirometry workshops since a one-day seven-hour effective knowledge and practical training would most likely have significant impact on participants’ spirometry practice with its expected positive outcome on respiratory health in Nigeria.

## Background

The burden of respiratory diseases in Nigeria is enormous [1] with varying published prevalence of respiratory illnesses that have lung-function complications. These include pneumonia and its various complications, tuberculosis [2], asthma [3], chronic obstructive pulmonary disease (COPD) [4] as well as diseases that have lung-related co-morbidities like sickle cell anemia (SCA) [5], and HIV [6] which have attendant restrictive (interstitial) or obstructive residual effects on the lung. Furthermore, associated environmental inhalations that affect the lung, such as environmental tobacco smoke exposure [7], use of biomass fuels for cooking [8], gas flaring activities, and air pollution [9] also abound in Nigeria. Regarding the ability to diagnose their effect on the lungs and thus make policies that could reduce associated morbidities, it is important that the health worker be versed in the use of lung-function assessment tools like spirometry. Furthermore, other equipment for lung-function assessment (plethysmograph, gas diffusion/dilution measurements, fractional exhaled nitric oxide FeNO equipment), due to cost, are not readily available in Nigeria, while the more available peak flow meters, have diagnostic limitations. The Computed tomography of the chest (Chest CT), is expensive and not routinely requested. Where competency for the use of spirometer is enhanced, respiratory disease burden can at least be objectively quantified and referrals made appropriately to pulmonologists [3, 10]. Several studies have reported a global inadequacy in the knowledge and use of spirometry for the diagnosis and treatment of respiratory diseases such as COPD and asthma [11].

Although spirometry is relatively easy to carry out, its clinical utility is however partly dependent on the competency and knowledge of the personnel performing and interpreting the test results [12–16]. Where the necessary competency for the use of spirometer is lacking, disease burden cannot be objectively quantified with resultant misdiagnosis and mismanagement of such respiratory diseases like asthma and COPD, as inappropriate interpretation of spirometric values would mean a different diagnosis and a different treatment pathway [17]. In other instances, patients with dyspnea and wheezing have been diagnosed with cardiac diseases leading to people in low-and middle-income countries (LMIC), spending scarce resources on investigations like 2-D Echo and diagnosis of asthma eventually confirmed after proper spirometry is done [18]. Other asthma mimics have also been correctly diagnosed by deploying spirometry [19]. Furthermore, competency in spirometry will also ensure detection and reduction in disease-related morbidity such as in children with HIV or SCA where early lung-function assessments during childhood identify early those who require long-term follow-up. Studies have therefore highlighted the need to improve spirometry knowledge and training through spirometry workshops, as well as improve quality assurance to meet acceptable standards [20–22].The best approach would also include assessing competence at end of training which should include both knowledge base and skills acquisition assessment, done in stages [21, 22]. The appropriate use of the spirometer will therefore requires quality training of the health personnel. In both private and public health facilities, spirometry performance is not usually limited to spirometry technicians [23].On the contrary, in Nigeria and other LMIC, the test procedure is carried out by doctors, nurses, and other health workers who sometimes may not have received any formal spirometry training. In the developed world like the United States, technicians who perform spirometry test compulsorily complete a nationally approved course with at least a minimum of 3-yearly refresher courses [20, 21, 24]. In New Zealand, Australia, and South Africa, training courses with international accreditation for spirometrists or anyone who does reliable spirometry also exists [20, 25, 26]. The American Thoracic Society (ATS)/European Respiratory Society (ERS) recommend that spirometry should be performed by trained, competent, and experienced personnel. They should be able to correctly teach participants how to assess the correct performance of the spirometry test by patients and the quality of the test result and should form the core of the training team [21].There has been a few formal training of Nigerian health workers on spirometry, most of which have been with the collaboration of funded international agencies like Pan-African Thoracic Society (PATS)/MECOR courses and Breathe Africa. Similarly, a South African training body-Spirometry Training Services Africa (STSA) has held several spirometry courses and workshops in many parts of Africa [26], and recently undertook a training course in South-West Nigeria. No such course has taken place in South-East Nigeria and other regions of Nigeria, and there are no known regular formal indigenous training courses with re-certification opportunities in Nigeria. The Little Lung Africa respiratory team, made up of trained and certified spirometrists (see attached supplementary file), thus undertook a one-day hands-on spirometry training workshop, with testing of knowledge following theory and hands on session (phase 1) and a future specific skills assessment planned (phase 2). The team used a similar curriculum as the ERS and STSA [21, 24, 26] to create a ripple effect on the knowledge and application of spirometry and to evaluate the impact of this limited one-day intervention, while showing the need for a funded re-certification course domiciled in Nigeria.

## Methods

This prospective study was descriptive and cross-sectional. The study population was health workers of various cadres who participated in the spirometry course at Little Lung Africa respiratory center Enugu and the Pre-conference workshop of the Nigerian Thoracic Society in Calabar Nigeria, in August and November 2019 respectively. Qualified and experienced resource faculty members, who had received formal certified training in spirometry served as facilitators for the courses in both cities. Spirometry certifications of the various resource persons included those received from Queen Mary University London/Barts and the London Hospital; Red Cross Children Hospital/University of Cape Town, South Africa; European Respiratory Society/Summer school of ERS; ERS Drivers license spirometry course, PATS-MECOR spirometry course (headed by the STSA team). The resource persons who served as faculty for the course are leading respiratory teams in their various hospitals where they also carry out lung function tests in children and adults on regular basis. These certified resource persons had together produced a curriculum adapted from ERS curriculum and similar to the trainings they had all previously received and thus formed a harmonized curriculum for this training with similar methods from the various certifying bodies (Supplementary file 1). The curriculum included topics on basic lung anatomy and mechanics theory for spirometry, the various types of spirometry equipment, indications and contraindications for spirometry, the test procedure, standard criteria for acceptability and repeatability, interpretation of results of spirometry, quality control and calibration of the instrument. Bronchodilator reversibility testing was also part of the curriculum. Training on advanced spirometry, such as bronchial challenge testing with methacholine, was not part of the core MECOR, STSA, or ERS training curriculum [24, 26], and thus was not included in this one-day training. Participants were self-funded and belonged to various health working cadres and with varying experience and previous spirometry exposure. They were taught didactic lectures on various aspects of spirometry for three hours. Prior to the lectures, a knowledge-based written pretest was conducted and following the lectures on the same day, hands-on practical sessions were then taught by the qualified resource persons and subsequently practiced by all participants for another four hours. The equipment used included three ndd easy one® ultrasound-based spirometer, three pneumotach rotating vane spirometer, and two 3 *l* calibration syringes. Participants were made to rotate in small groups of four participants in each group, rotating through five practical training stations where they performed supervised hands-on practice. The mounted stations were: spirometry performance, calibration, validation, and Global Lung Initiative (GLI) standardization of spirometry results and interpretation of spirometry tracing. For the performance of good spirometry station, the participants were taught to go through the given checklist for the procedure, position appropriately and then guide the patient to maximally inhale, forcefully exhale and keep going till all the air is possibly let out, before then performing an inspiratory maneuver, while verbally encouraging the patient all through the procedure [27]. The participants used each other to do real-life measurements and test interpretation and were also given actual printed out tracings from real patients with which to practice at that skill station. The spirometer types used for the training all had visual inter-phase screens which enabled the participants to see the flow volume loops made with each blow, and participants were taught real-time use of the ERS free online software for GLI conversion was utilized to standardize to age, height, gender, and race, including using the lower limit of normal (LLN) values. Participants were taught how to use their smart phones to access this software online as a ready-to-use office smart tool. Each participant practiced with both types of spirometers using syringes for the calibration checks. Care of the syringes and re-certification information for the syringes were also given to all participants. The participants’ knowledge on the various aspects of spirometry was pretested with a questionnaire before the commencement of lectures. The theory lectures were further reinforced with the practical hands-on sessions. Then, a written post-test of the acquired knowledge and skills, was conducted at the end of the course to assess if the one-day course improved their competency in spirometry. Participants willing to pay for and do examination for skills competence (phase 2) were informed of the opportunity. This was unfortunately interrupted by the pandemic. For homogeneous assessment of the training impact, the post-test questions used were exactly a copy of what served as the pretest questions. The questions consisted of topics from all aspects of the curriculum. Data of those who completed the tests were analyzed. Baseline data collected included age of participants, gender, specific profession, previous spirometry formal training, and the number of years since obtaining a first degree from a tertiary institution, years of professional practice, possession of spirometers or peak flow meters in the primary workplace, knowledge, and use of Global initiative for asthma (GINA) guidelines in asthma management. The normality of data was assessed, and mean knowledge scores for pretests and post-tests were calculated. The ANOVA statistics assessed for any difference between years of practice and test scores. The test of homogeneity of variance in test scores was assessed depending on available spirometers at the workplace of participating health workers. Outcomes were represented as tables. We adopted *p* < 0.05 as the level of statistical significance.

## Results

### Characteristics of study participants

**Fig. 1:**
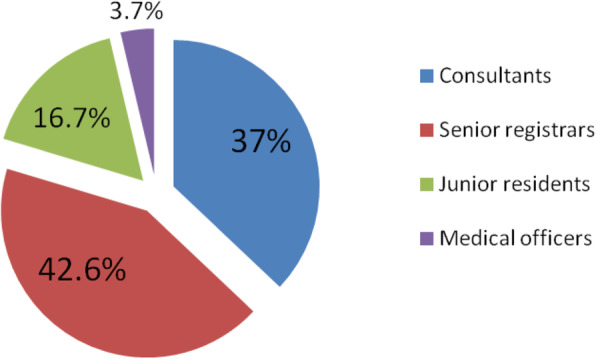
Various cadre of participating doctors

There were 64 participants with a mean age of 39.3 ± 6.0 years, of whom 54.7% (35/64) were females (Table 1). Of these participants, 82.8% (53/64) were medical doctors of varying cadre (Fig. 1), while the remaining 17.2% (11/64) were other health workers: laboratory technicians, nurse assistants, physiologists, and physiotherapists. For all participants, the mean number of years since obtaining a tertiary education was 12.8 ± 5.5 years (range 2 to 36 years), while the mean number of years practiced in chosen career differed slightly and was 9.9 ± 6.7 years. About 14.1% (9/64) had received formal spirometry training; another 15.6% (10/64) had received some informal training at their workplace, while 70.3% (45/64) had no previous exposure to spirometry training. Of all participants, 60.9% (39/64) knew of the existence of the GINA guidelines, which emphasizes the need for spirometry in asthma diagnosis and management. Fewer participants, 51.6% (33/64), attested to knowing the GINA guideline contents. There were 59.4% (38/64) who personally used various lung function equipment to support a diagnosis of asthma, of which 37.5% (24/64) used spirometers. In contrast, 21.9% (14/64) had access to only peak flow meters as a lung function tool. About half (56.3%) had been involved within the preceding three months in managing any patients with asthma (Table 1). Up to 40% did not have any lung function equipment domiciled at their place of practice (Table 2). The proportion of participants that reported possession of one or more spirometers at their place of practice was 60.9% (39/64), as shown in Tables 2 and 3, while 39.1% (25/64) had no spirometers and no other lung function equipment available (Table 4).

**Table 1:** Participants demographics, years of practice, spirometry training and asthma management

Variables	Outcome
Mean Age in years n (SD)	39.27 (6.02)
Years of graduation n (SD)	12.81 (5.49)
Years of practice n (SD)	9.90 (6.73)
**Gender** %(n)	
Male	45.3 (29)
Female	54.7 (35)
**Previous spirometry training** %(n)	
Formal training	14.1 (9)
Informal training	15.6 (10)
None	70.3 (45)
**Knowledge of the existence of GINA**%(n)	
Yes	82.8 (53)
No	17.2 (11)
**The practice of GINA contents** %(n)	
Yes	51.6 (33)
No	48.4 (31)
**Uses of any lung function equipment to support asthma diagnosis**%(n)	
Yes	59.4 (38)
No	40.6 (26)
**Recent management of the patient with asthma**%(n)	
Less than three months	56.5 (36)
More than three months	43.8 (28)

**Table 2:** Availability of spirometers in places of practice

Spirometer available	Frequency	Percent
No	25	39.1
Yes	39	60.9
Total	64	100.0

**Table 3:** Number of spirometers in place of practice

Number of Spirometers available	Frequency	Percent
0	25	39.1
1	22	34.4
2	12	18.8
3	5	7.8
Total	64	100.0

**Table 4:** Type of Lung function equipment available in places of practice

Lung function equipment available	Frequency	Percent
None	26	40.6
Peak flow meter	14	21.9
Spirometer	24	37.5
Total	64	100.0

### Knowledge test results

Fifty participants had complete test scores, and these were further analyzed. The mean pre-lecture percentage test (SD) score was 52.95 (24.14) %, with scores ranging from 11.86 to 91.0%, while the mean post-test score was 64.61 (16.84) %; score range 28.00 to 91.53% (Table 5). There was a vast knowledge score variance of 582.72 among the diverse participants who became less variable (283.51 (r = 0.76; *p* < 0.001) after knowledge impartation via lectures and hands-on training had occurred.

**Table 5:** Knowledge test scores of all participants (before and after theory/practice sessions)

Test score (%)	Mean (SD)	95%CI	*P* value
Pre-training	52.97 (24.38)	46.04–59.89	< 0.001
Post-training	64.72 (16.99)	59.84 69.60	

The number of years of practice did not affect test scores (Table 6), but the pretest knowledge scores were affected by the year of graduation (*p* = 0.02), as shown in Table 6. This effect was no longer seen at the post-test after the training session had occurred (*p* = 0.39), as displayed in Table 7.

**Table 6:** Factors affecting knowledge outcome scores

Factors affecting test scores	Mean square of test scores	F	***P***-value
**Years of practice**			
Pre-test score	651.27	1.18	0.34
Between Groups	552.11		
Within Groups			
Post-test score	341.79	1.35	0.23
Between Groups	253.89		
Within Groups			
**Year of graduation**			
Pre-test score	926.44	2.44	**0.02**
Between Groups	379.24		
Within Groups			
Post-test score	301.05	1.107	0 39
Between Groups	271.88		
Within Groups			
**Total number of spirometers available at practice centers (0 to 3)**			
Pre-test score	310.38	0.51	0.68
Between Groups	613.14		
Within Groups			
Post-test score	683.92	1.107	0 39
Between Groups	271.88		
Within Groups

**Table 7:** Effect on the year of graduation following training

Test scores		Sum of Squares	df	Mean Square	F	Sig.
**Pretest** score	Between Groups	15,749.398	17	926.435	2.443	**.015**
	Within Groups	11,756.311	31	379.236		
	Total	27,505.709	48			
**Posttest** score	Between Groups	5418.862	18	301.048	1.107	.394
	Within Groups	7612.519	28	271.876		
	Total	13,031.380	46			

## Discussion

The present study has demonstrated that only 14.1% of participants had received prior formal training on spirometry. Studies [15, 28–30] have shown that there is a lack of formal training in spirometry worldwide and that it is not just a problem of resource-limited countries, although such countries are worse off. In a questionnaire-based study in Australia, where 5976 general practitioners were asked details of spirometer ownership, usage and the level and source of spirometry training, they reported that only 42% of the respondents had spirometers in their practices [31]. Furthermore, 77.8% of the practices never checked spirometer accuracy using a calibration syringe. In 38.2% the spirometer training courses were usually less than 2 h and were organized by general practitioners [31]. This would inadvertently affect the quality of spirometry data produced from the various hospitals run by the doctors. Schermer and co-workers [15] found that only 39% of tests performed in general practices in the Netherlands met acceptability and repeatability criteria. They suggested such reasons as limited training, quality assurance activities, and lack of experience as responsible for the high rate of low-quality spirometry tests observed in general practice. Borg and colleagues [13] observed that of the participants who were unable to meet acceptability and repeatability criteria at follow-up assessments at 5 and 7 months after training, most of whom had not continued to practice spirometry. The present study was a one-day course that assessed the participants based on the outcome of their training following full-day hands-on workshop and possibly could not indicate if participants continued the practice: especially for the participants who did not have any spirometers domiciled in their place of practice. In a mixed-methods study [10] that compared models of spirometry delivery in primary-care settings for patients at risk of COPD, the authors found that spirometry performed by visiting trained nurses was of a higher quality than spirometry performed by nurses or general practitioners (GP) from the practice (76% vs. 44%). In our study, the majority of our participants were medical doctors, and there was a wide variation in its homogeneity of pre-training scores among all participants. We noted from the scores in pre-test that the years of graduation significantly reflected in the score before knowledge was imparted and that this effect was no longer present at the post-test after everyone had been trained, irrespective of the profession, years of practice, or years of graduation.

Our study has shown that the majority had this unmet need of limited or no training on spirometry irrespective of profession. Similarly, another study [16] investigating spirometry use in general practice among doctors in a Welsh community, found that 66% of General Practitioners had limited or no confidence in interpreting results. This finding was in consonance with another survey where among General Practitioners in the Netherlands, 69% felt that they required ongoing support with the interpretation of spirometry [31]. Another study in the United States demonstrated a 76% concordance between a GP and a respiratory expert in the interpretation of spirometry results [32].All these findings buttress the fact that training the spirometer operator and the reporter/interpreter is important for providing a quality spirometry service irrespective of the profession. Furthermore to maintain a good level of competency in spirometry it is remains important to encourage participants to go ahead and specifically do a skills competency assessment, as was planned in this training before the onset of the COVID-19 pandemic.

The present study does however report an increase in knowledge after one-day spirometry training. This observation is similar to findings reported in other intervention studies. In these studies, knowledge, practice, acceptability, and repeatability improved in both training types: less than one day and the one day or more spirometry trainings, over a year with ongoing feedback training [12, 14, 17, 33–35] In a mentorship based intervention study by Gupta et al. conducted over 12 months; there was a significant improvement in knowledge score post-intervention across all health workers that participated in that survey. Furthermore, Eaton et al. [12] in another intervention study in New Zealand, reported a significant training effect between participants who had a one-day training and those who had no training (*p* < 0.0001). Those who had been trained became better skilled to identify normal spirometry results and to provide spirometry measurements as a screening and monitoring tool [12]. Our finding, however, differed from that by Borg et al. [13] in a study in Australia where a 14-h spirometry training did not provide the skill to perform spirometry according to the ATS criteria and suggested the need for follow-up training. Their finding may be related to the small sample size used. Another difference could have arisen from the proportion of participants who had already undergone previous spirometry training, underscoring the fact that change in the acquired knowledge may not be significant, unlike the current study where majority had no formal training. Parsons and co-workers [33] also reported that in as much as knowledge improved generally among participants who had completed a 20-week training including online and face-to-face workshops, in those participants who had undergone previous spirometry training there were notable better knowledge and skills in spirometry measurements and interpretation. This further buttresses the need for spirometry training for health care workers as done in this study. Participants who eventually return for part 2 of the skills assessment would probably benefit the most from such a training course.

### Study limitations

The length of a spirometry training course, follow-up training, and quality feedback are important inclusions of a training programme to improve the quality of spirometry results. Some of these components were lacking in this study as it was limited to a one-day course that focused on the effect on the knowledge base (theory and practical) pretests and post-tests following effective training and did not test the effect on skill, competence and whether it had truly improved among the participants. The planned phase 2 of the course was unfortunately, significantly impacted by the COVID-19 pandemic. However, certified resource persons ensured that in this part 1 course, each of the four students that rotated through each station had enough time to practice skills impacted but did not set a separate skills station exam that was scored. Due to limited funds to further host a certification of skill course, we have not yet had the opportunity to test for repeatability in the providers. It would also have required more than a one-day workshop to assess this outcome and has been built into future part 2 course. The specific aspects of knowledge, if analyzed separately, may have informed aspects that may require greater reinforcement, but this has been built into a skill competency-training course that assesses what skills will require to be taught with emphasis. The effect of one-day 7 h training, however, remains remarkable despite these limitations.

## Conclusions

The present study has shown that a one-day spirometry workshop significantly improved the knowledge of spirometry practice. The need to set up more frequent locally organized spirometry workshops as even a one-day practical training would most likely have a significant impact on participants’ spirometry practice with its expected outcomes. Such interventions aimed at improving the competencies of health workers are therefore recommended.

## Supplementary information

**Additional file 1.**

## Data Availability

The minimal dataset that would be necessary to interpret, replicate and build upon the findings reported in the article are available with the corresponding author. The datasets generated during and/or analysed during the current study are not publicly available due to some contents that are private but are available from the corresponding author on reasonable request.

## Abbreviations

ANOVA: Analysis of variance
ATS: American Thoracic Society
COPD: chronic obstructive pulmonary disease
CT: Computed tomography
ERS: European Respiratory Society
FeNO: Fractional exhaled Nitric oxide
GINA: Global initiative on asthma
GLI: Global Lung Initiative (GLI)
HIV: Human immunodeficiency virus
LLA: Little Lung Africa
LLN: Lower limit of normal
LMIC: low-and middle-income countries
MECOR: Methods in Epidemiologic, Clinical and Operations Research
NTS: Nigerian Thoracic Society
PAT: Pan-African Thoracic Society
SCA: Sickle cell anemia
STSA: Spirometry Training Services Africa
