# Supplementary material for: Spirometry practice and the impact of a phase 1 training workshop among health workers in southern Nigeria: a cross-sectional study
Source: BMC Pulm Med. 2020 Oct 6;20:258. doi: 10.1186/s12890-020-01291-8 (PMC7538053; doi:10.1186/s12890-020-01291-8)
Supplement: Supplementary file 1 — Additional file 1. [file 12890_2020_1291_MOESM1_ESM.pdf]

Spirometry training course

…raising an army of spirometrist
(the RIPPLE effect)

SPIROMETRY TRAINING COURSE (LITTLE LUNG AFRICA) FACULTY

TRAINERS AND QUALIFICATIONS

- **DR ADAEZE AYUK** – *CONSULTANT PEDIATRICIAN PEDIATRIC PULMONOLOGIST (UCT) - SPRIROMETRY TRAINING -SOUTH AFRICA RED CROSS CHILDREN HOSPITAL/ CMSA; BARTS & THE LONDON HOSPITAL/QMUL; PATS MECOR TRAINING; ERS TRAINING – SUMMER SCHOOL; LEVEL 4 ERS DRIVERS LISCENCE*
- **DR CHIZALU NDUKWU** - *CONSULTANT PEDIATRICIAN; ERS SPIROMETRY DRIVERS LISCENCE; PATS MECOR TRAINING*
- **DR NNAMDI NWOSU** – *CONSULTANT RESPIRATORY PHYSICIAN; PATS MECOR SPIROMETRY TRAINING*
- **DR JOY EZE -***CONSULTANT PEDIATRICIAN;; PATS MECOR TRAINING*

Basis of the Training programme content (adapted from ERS)

Phase 1: Knowledge and basic skills

- Teaching of theoretical course and completion of written tests including multiple-choice question assessment of the course and practical aspects taught

Phase 2: Knowledge and competence in measurement

- It is recommended that participants complete this part within 3 - 12 calendar months of completing Part 1 and includes the completion of a training course which will focus on competency based training on practicing spirometry.

GENERAL CURRICULUM

- **Part 1: Knowledge and basic skills AND Knowledge of competence in measurement (with supporting practice sessions)**
- Basic Lung Anatomy and Mechanics
- **Spirometry** Equipment.
- **Spirometry** Indications and Contraindications.
- Test Procedures.
- Criteria for Acceptability and Repeatability.
- Reversibility Testing. Office Procedures and Basic Interpretation.
-

  Theory for spirometry
- Indicationsfor test
- Instructionsfor test
- Contraindications of test
- Conduction ofspirometry
- Interpretation of resultsof spirometry

Outline(Phase 1)

- General overview of spirometry
- Indications/contraindications
- Preparing the patient and equipment
- Spirometry procedure/recognition of poor quality manoeuvres
- Reference equations
- Understanding the spirogram;
- Interpreting spirometry; Normal/abnormal results
- BDR testing
- Quality control/quality assurance
- Practice sessions

PRACTICE SESSION

- Physiology of lung function testing and spirometry
- Indications and contraindications for spirometry
- Instructions for test
- Conduction of spirometry and quality control including calibration
- Interpretation of results of spirometry including real life spirograms
- BDR testing

General overview of spirometry

Physiology

Lung Volume and Subdivisions

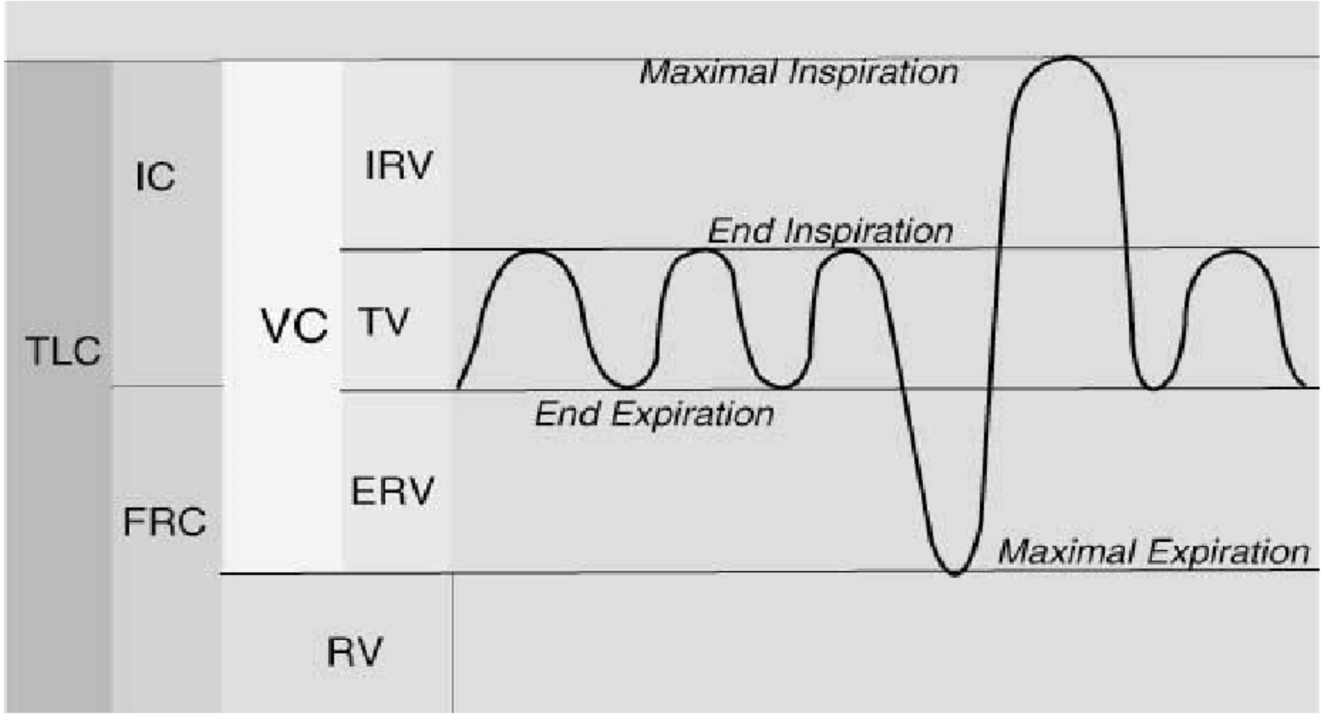

What **lung function** measurements check

- Lung volumes - How much air volume can be moved in and out of the lungs
- How fast the air in the lungs can be moved in and out (flow)
- How stiff are the lungs and chest wall (lung compliance)
- The diffusion characteristics of the membrane through which the gas moves (oxygenation)
- How the lungs respond to chest physical therapy procedures

Some **indications** for Pulmonary Function Testing

- Screening for the presence of obstructive and restrictive diseases
- Evaluating the patient prior to surgery especially if patients have:
  - ❑ pulmonary disease
  - ❑ pathologically obese
  - ❑ history of smoking, cough or wheezing
  - ❑ will be under anaesthesia for a lengthy period of time
  - ❑ are undergoing an abdominal or a thoracic operation
- Evaluating the patient's condition for weaning from a ventilator.
  - If the patient on a ventilator can demonstrate a vital capacity (VC) of 10 - 15 ml/Kg of body weight, it is generally thought that there is enough ventilatory reserve to permit weaning and extubation.
- Documenting the progression of pulmonary disease restrictive or obstructive
- Documenting the effectiveness of therapeutic intervention
- Epidemiological surveys

Different **lung function** measuring techniques

- Spirometry
- Plethysmography
- Transfer factor (TLCO or DLCO)
- Forced Oscillation Technique
- Peak flow meter(PFM)
- Gas dilution methods

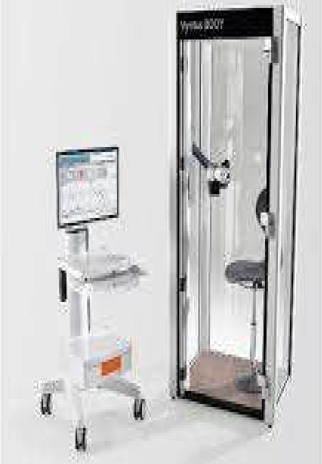

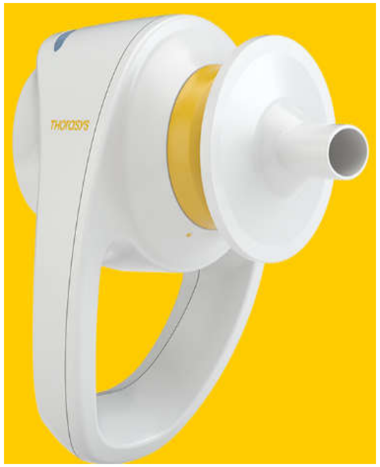

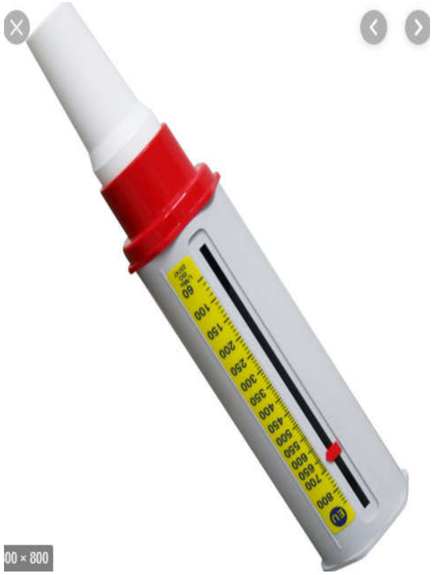

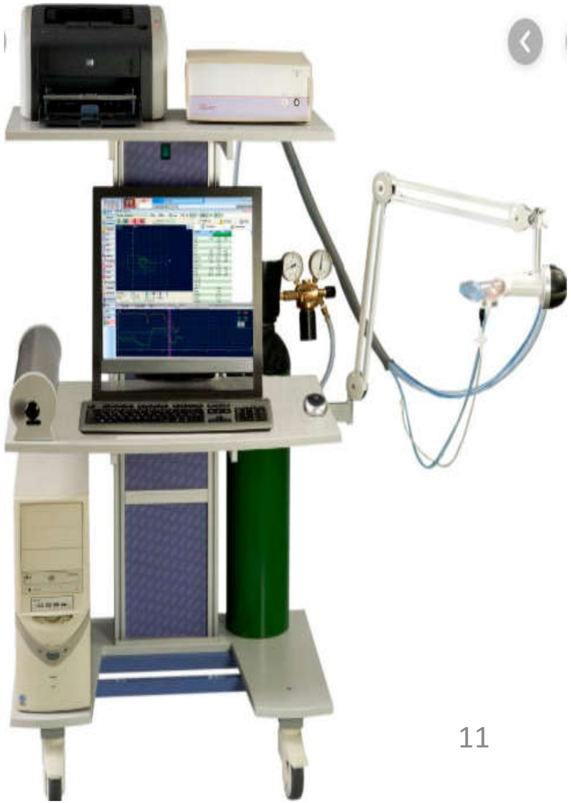

SPIROMETRY
spiro – breathe; **meter** – to measure

- Spirometry is a physiological test that measures how an individual inhales or exhales volumes of air as a function of time.
  - - how much (volume) and how fast (flow)
- The primary signal measured in spirometry may be volume or flow.
- **Spirometry is invaluable as a screening test of general respiratory health in the same way that blood pressure provides important information about general cardiovascular health.**
- However, on its own, spirometry does not lead clinicians directly to an aetiological diagnosis

Volume displacement spirometers (older mechanics)

A

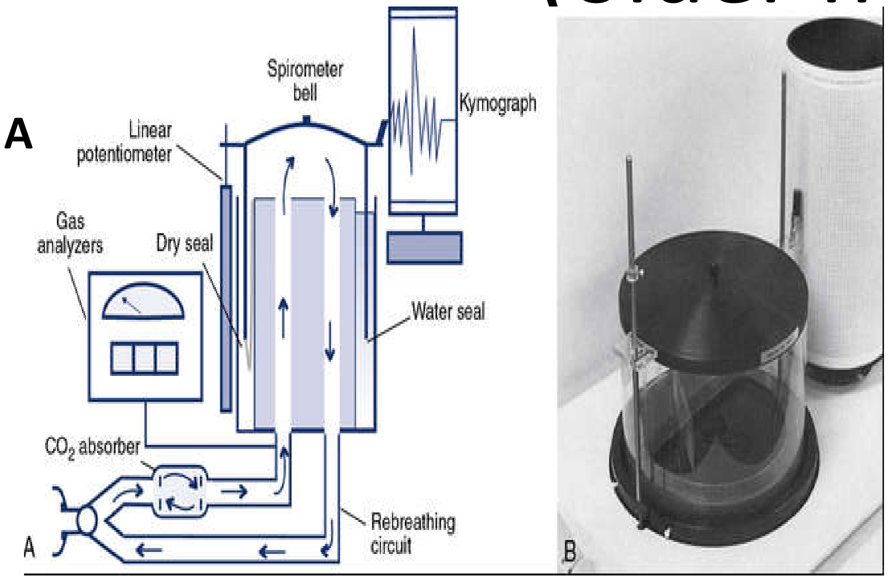

C

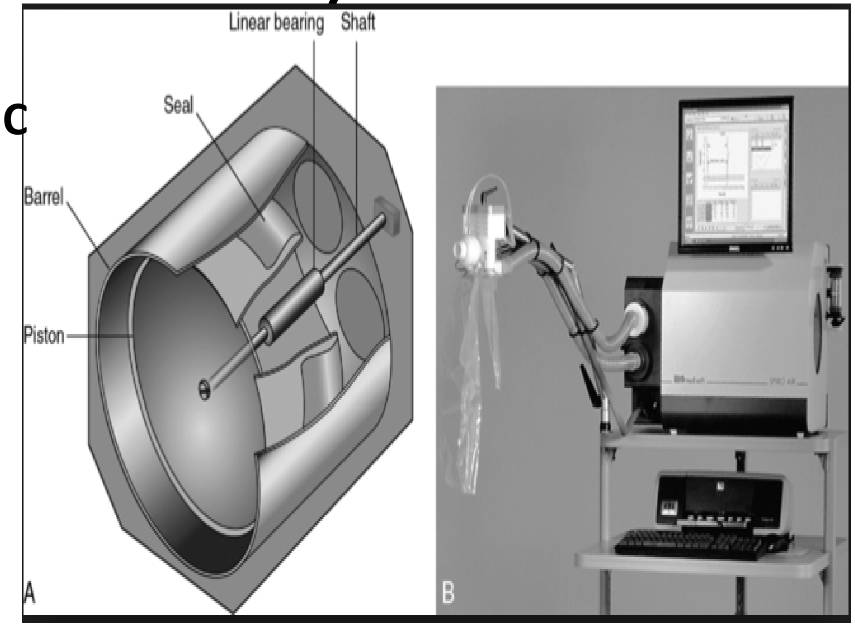

B

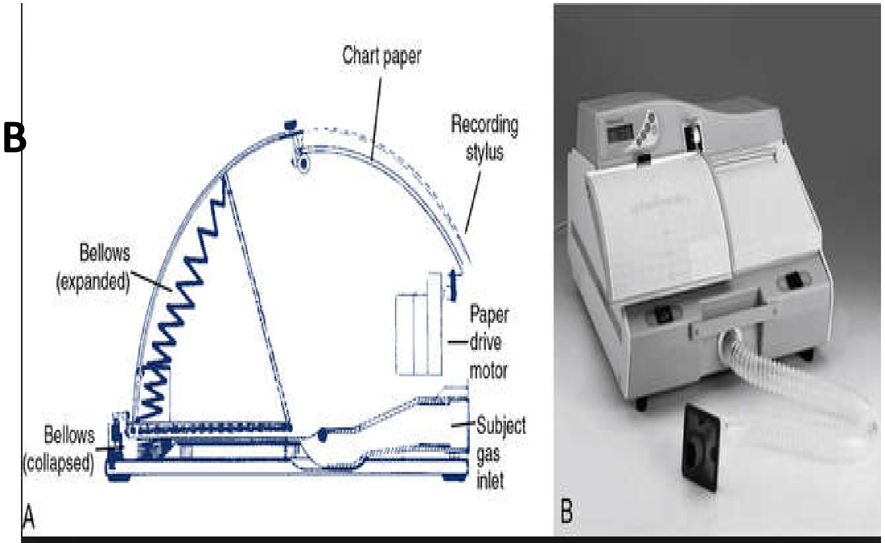

A. **Water-seal and Dry-seal spirometers**
B. **Wedge bellows spirometer**
C. **Dry rolling seal spirometer**

Various types/mechanics of spirometers –using FLOW

A. **Pressure**-differential **flow** sensors – pneumotachograph
B. **Heated** wires **flow** sensors – anemometers, thermistor
C. Pitot tube **flow** sensors
D. Electronic **rotating vane/turbine** flow sensor
E. **Ultrasonic** flow sensors

Flow sensing spirometers

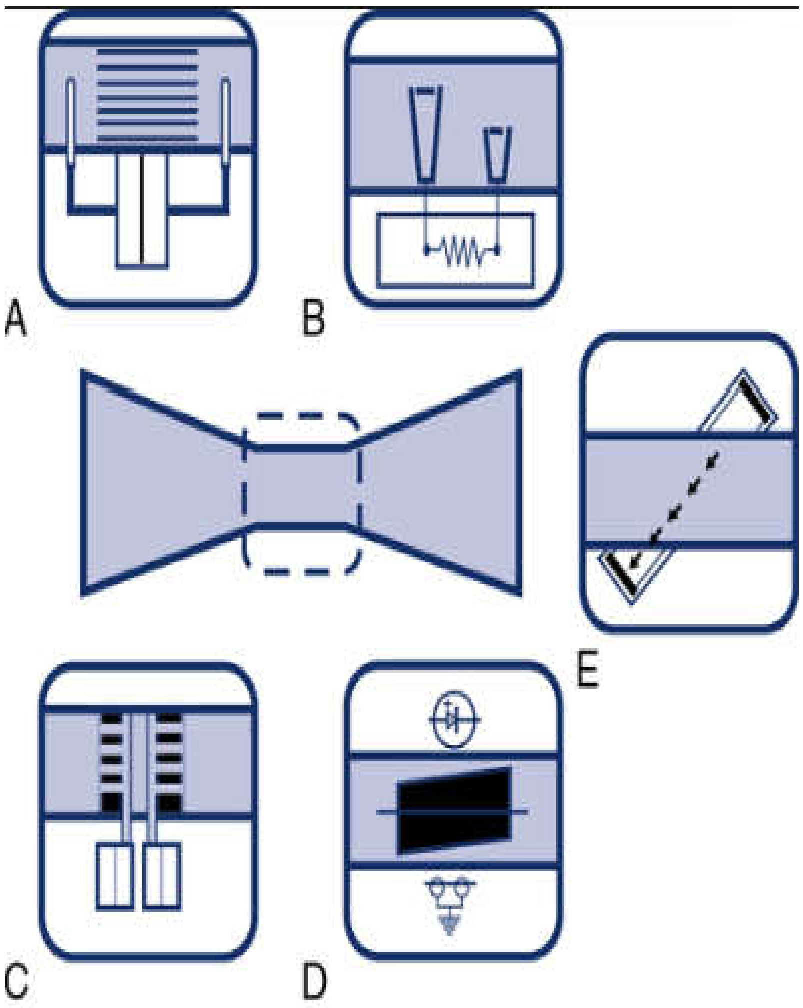

- **A. Pressure**-differential **flow** sensors – pneumotachograph
- **B. Heated** wires **flow** sensors – anemometers, thermistor
- C. Pitot tube **flow** sensors
- D. Electronic **rotating vane/turbine** flow sensor
- **E. Ultrasonic** flow sensors

Pressure differential spirometers

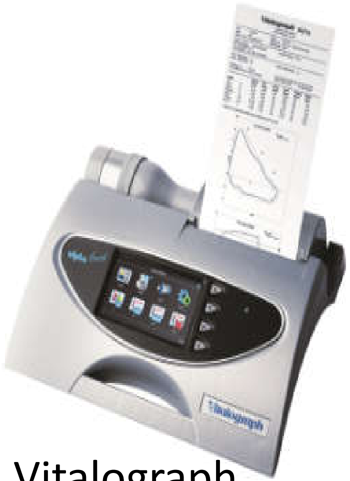

Vitalograph

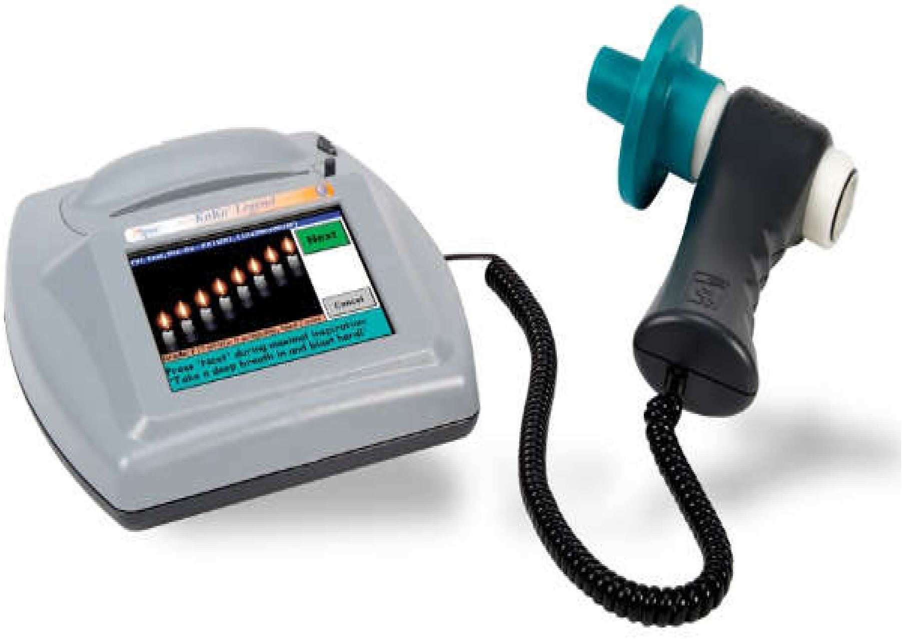

Koko spirometer

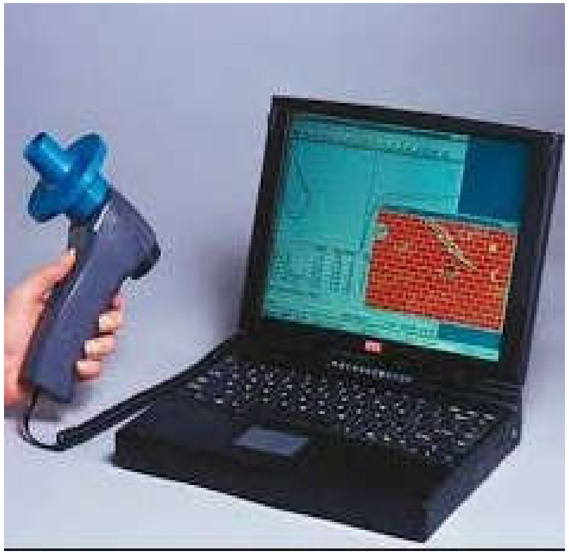

Koko spirometer

Turbine spirometers

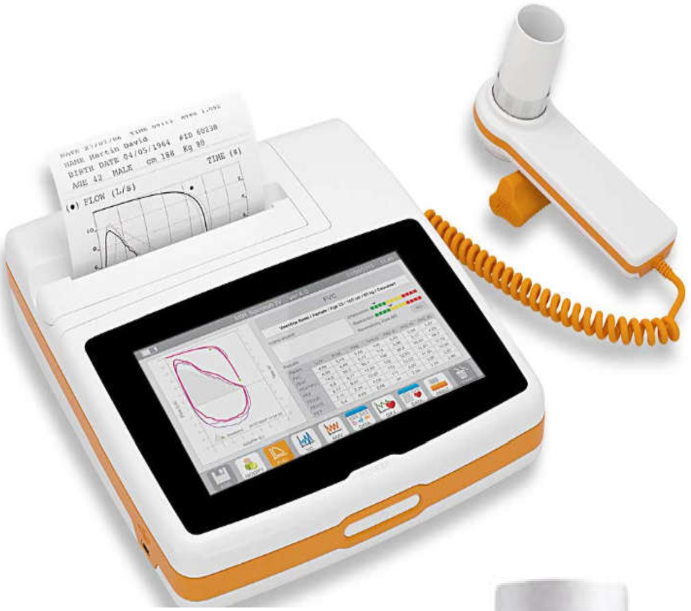

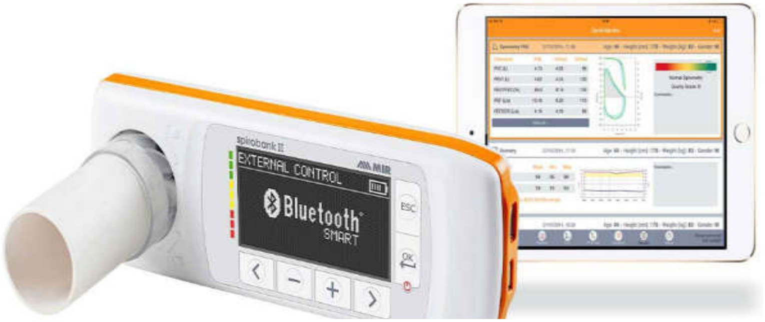

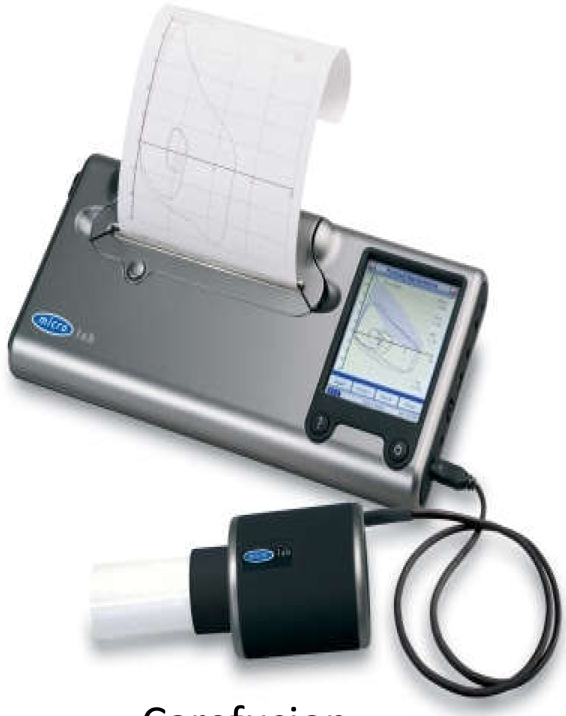

Spirolab

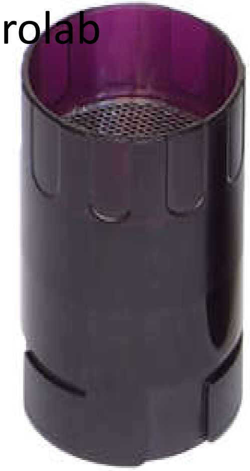

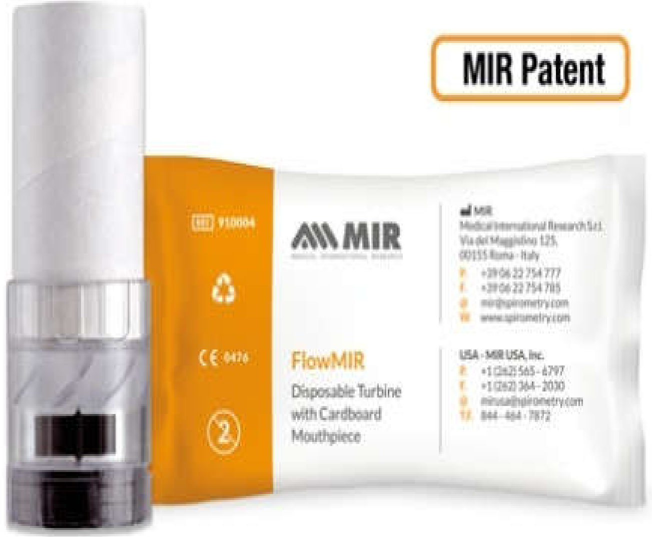

Carefusion

Ultrasonic spirometers

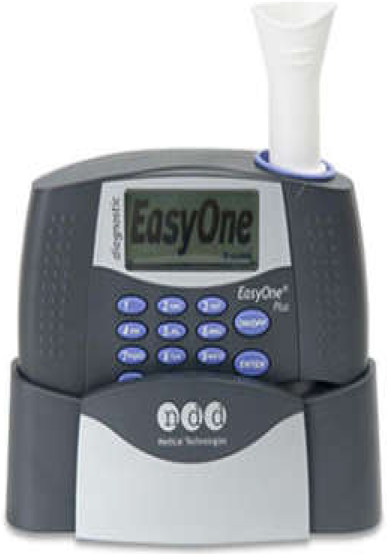

Easyone spirometer

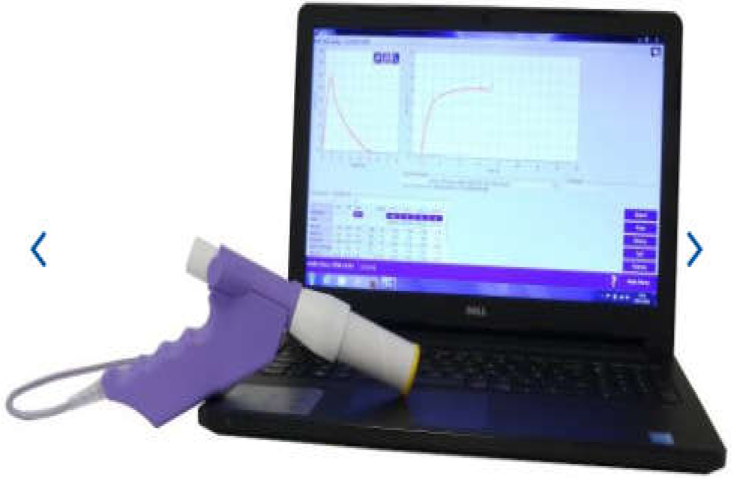

Easy-on spirometer with software

Why do Spirometry?

- Diagnosing
- Monitoring
- Disability evaluation
- Public health

Indications

Diagnostic

- To evaluate symptoms, signs or abnormal laboratory tests
- To measure the effect of disease on pulmonary function
- To screen individuals at risk of having pulmonary disease
- To assess pre-operative risk
- To assess prognosis
- To assess health status before beginning strenuous physical activity

Monitoring

- To assess therapeutic intervention
- To describe the course of diseases that affect lung function
- To monitor people exposed to injurious agents
- To monitor for adverse reactions to drugs with known pulmonary toxicity

Disability/impairment evaluations

- To assess patients as part of a rehabilitation programme
- To assess risks as part of an insurance evaluation
- To assess individuals for legal reasons

Public health

- Epidemiological surveys
- Derivation of reference equations
- Clinical research

What are the most important measurements?

- **FVC:** Forced Vital Capacity is the maximal volume of air exhaled with maximally forced effort from a maximal inspiration (expressed in litres)
  - volume delivered during an expiration made as forcefully and completely as possible starting from full inspiration
- **FEV1**: Forced Expiratory Volume in the first second (FEV1) is the maximal volume of air exhaled in the first second of a forced expiration from a position of full inspiration (expressed in litres)
  - the volume delivered in the first second of an FVC maneuver

FEV1/FVC

Ratio of FEV1 to FVC

- it indicates what percentage of the total FVC was expelled from the lungs during the first second of forced exhalation
- This value is critically important in the diagnosis of obstructive and restrictive diseases.
- It is expressed as percent (%)

Other parameters and what they mean

- PEF
- FEV6
- FEF 25
- FEF 50
- FEF 25-75
- FEF 75
  - (difference between FEF and MEF)
  - Maximal instantaneous forced expiratory flow MEF 75 (when 75%is remaining) is identical to FEF 25, which is maximal instantaneous forced expiratory flow where 25% of the FVC has been expired.
- BEV
- EOT

Equipment needed for Spirometry

- The operator (certified every 3 years)
- The cooperative patient – patient preparation
- The right environment/weather
- Weather meter
- Spirometer
- Calibration syringe
- Weight/height scale
- Mouth piece/nose clip
- Filter/facemask
- Waste bag
- pMDI/spacer
- BP machine

Performing spirometry

- Apply a nose clip to the patient's nose (this is recommended but not essential – be consistent)
- Urge the patient to: – Breathe in fully (must be absolutely full)
- Seal his/her lips around the mouthpiece
- Immediately blast air out as fast and as far as possible until the lungs are completely empty
- Breathe in again as forcibly and fully as possible (if inspiratory curve is required)
- Repeated severally until 3 identical efforts are recorded
- Bronchodilator given and test repeated (if required)

- To ensure an acceptable result, the FVC manoeuvre must be performed with maximum effort immediately following a maximum inspiration.
- It should have a rapid start and the spirogram and flow-volume curve should be a smooth continuous curve.
- To achieve good results, carefully explain the procedure to the patient, ensuring that he/she is sitting erect with feet firmly on the floor
- Use the most comfortable position for the patient, though standing gives a similar result in adults.
- Constant verbal encouragement required – “keep going”

In children

- Children give more consistent results sitting, and chances of syncope from effort, reduced.
- Need for demonstration
- Need for practice with balloon (and the peak flow meter)
- Need for incentive spirometry (has drawbacks)

Ensure ….

- There are no leaks
- No cough (note FEV1 may be valid if cough occurs after the first second)
- No glottis closure (Valsalva)
- No obstruction of the mouthpiece (e.g. by the tongue or teeth)
- No evidence that the patient took an additional breath during the expiratory manoeuvre
- Keep going until a plateau is seen
- Use very vigorous effort right from the start of the manoeuvre and continuing until absolutely no more air can be exhaled
- No leaning forward during the test
- Obtain at least 3 acceptable tests that meet repeatability criteria.

Spirometry preparations- before you start

- Patient data into the machine
- BPTS
- Reference equations choice
- Other parameters (configuration)
- Choice of best trial/best value

Acceptability Criteria

- The patient must have followed instructions
- A continuous maximal expiratory manoeuvre throughout the test (i.e. no stops and starts) should be achieved initiated from full inspiration
- No evidence of hesitation during the test/no slow start
- Test was performed with a **rapid** start
- PEF has a **sharp** rise (flow-volume)
- No **premature termination**, i.e. expiration continued until there was no change in volume
- The patient had blown for ≥3 seconds/plateau achieved (children aged <10 years) or for ≥6 seconds (≥10 years).
- FET-…………6 sec/plateau
- BEV -……A BEV > 150 mL or PEFT > 120 ms indicates a **slow** start.
- EOTV- 0.05L in last 2 secs

REPEATABILITLY CRITERIA – 3 tests/blows within 0.15L of each other (0.1L if FVC <1L)

Acceptability criteria

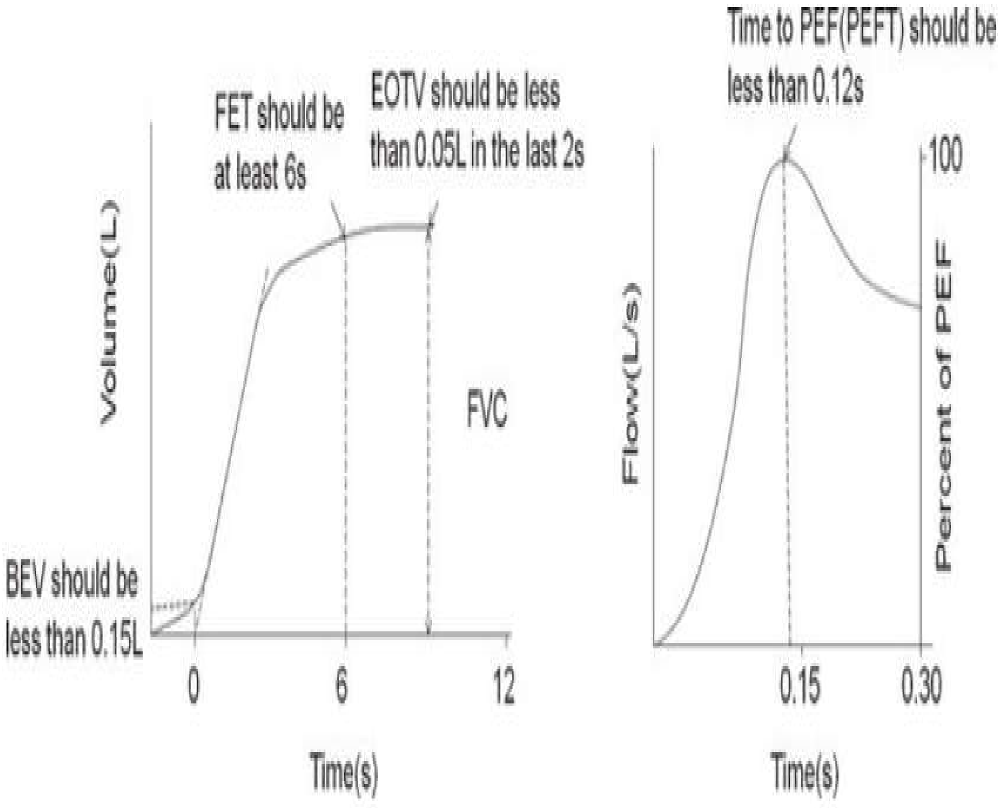

Figure 1. Acceptability Criteria [BEV, PEFT, FET, EOTV] for Spirometry tracings depicted in schematic drawings of Volume vs time and Flow vs time curves. BEV = back extrapolated volume; PEFT = time to peak expiratory fl ow; FET = forced expiratory time; EOTV = end of test volume as indication of presence of plateau.

This figure was uploaded by Kenneth R Chapman

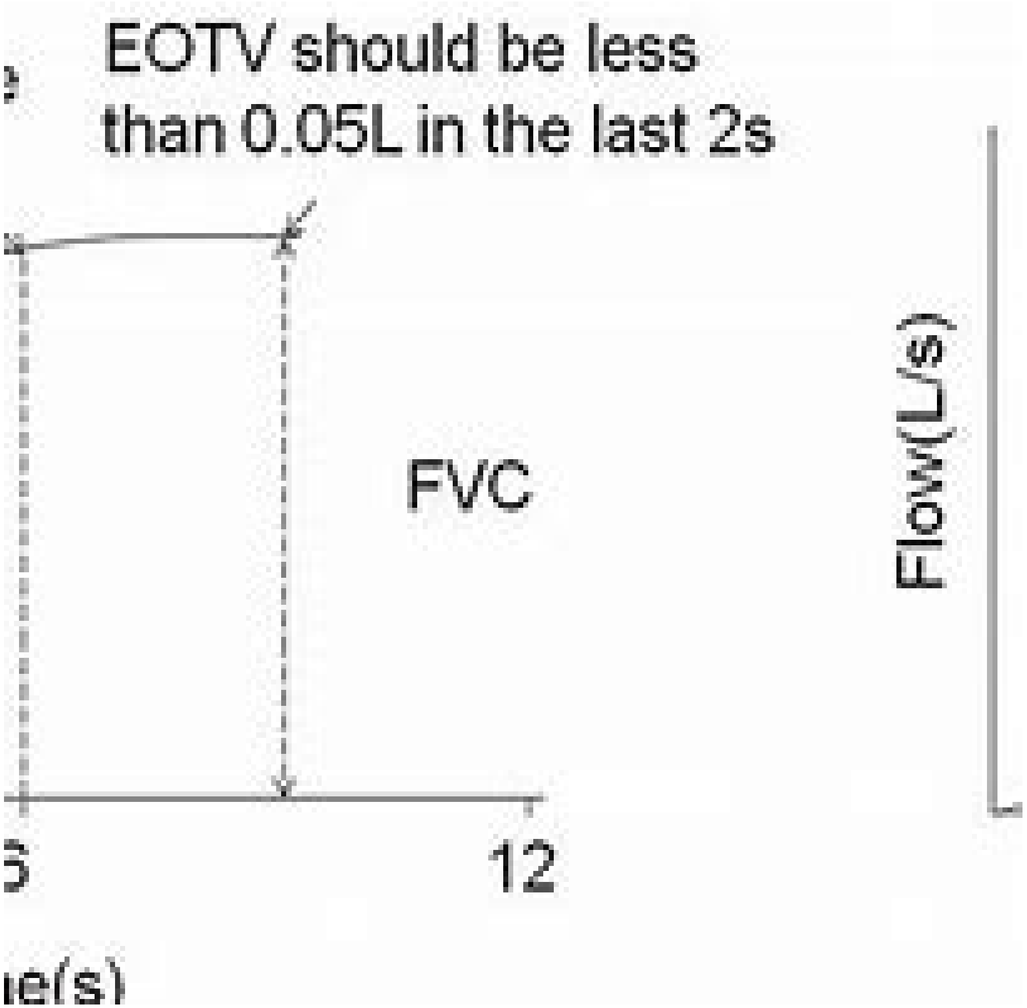

Nb: In CURRENT GUIDELINES, end of test criteria (EOT) no longer requires a patient to have achieved an expiration time of 3 or 6 seconds (depending on age) but rather that there is less than 0.0025L change in volume for at least 1 second

UNDERSTANDING DIAGRAMS IN SPIROMETRY

Diagrammatic representation of spirometry

- **Spirogram:** A spirogram is a **volume-time** curve, flow measured over a time period, usually 6 seconds
- **Flow-Volume curve**: Measures of **flow** can be made as a function of volume, thus generating a flow-volume curve ,
  - the shape is reproducible for any individual (signature) but varies considerably between different lung diseases.

Spirogram

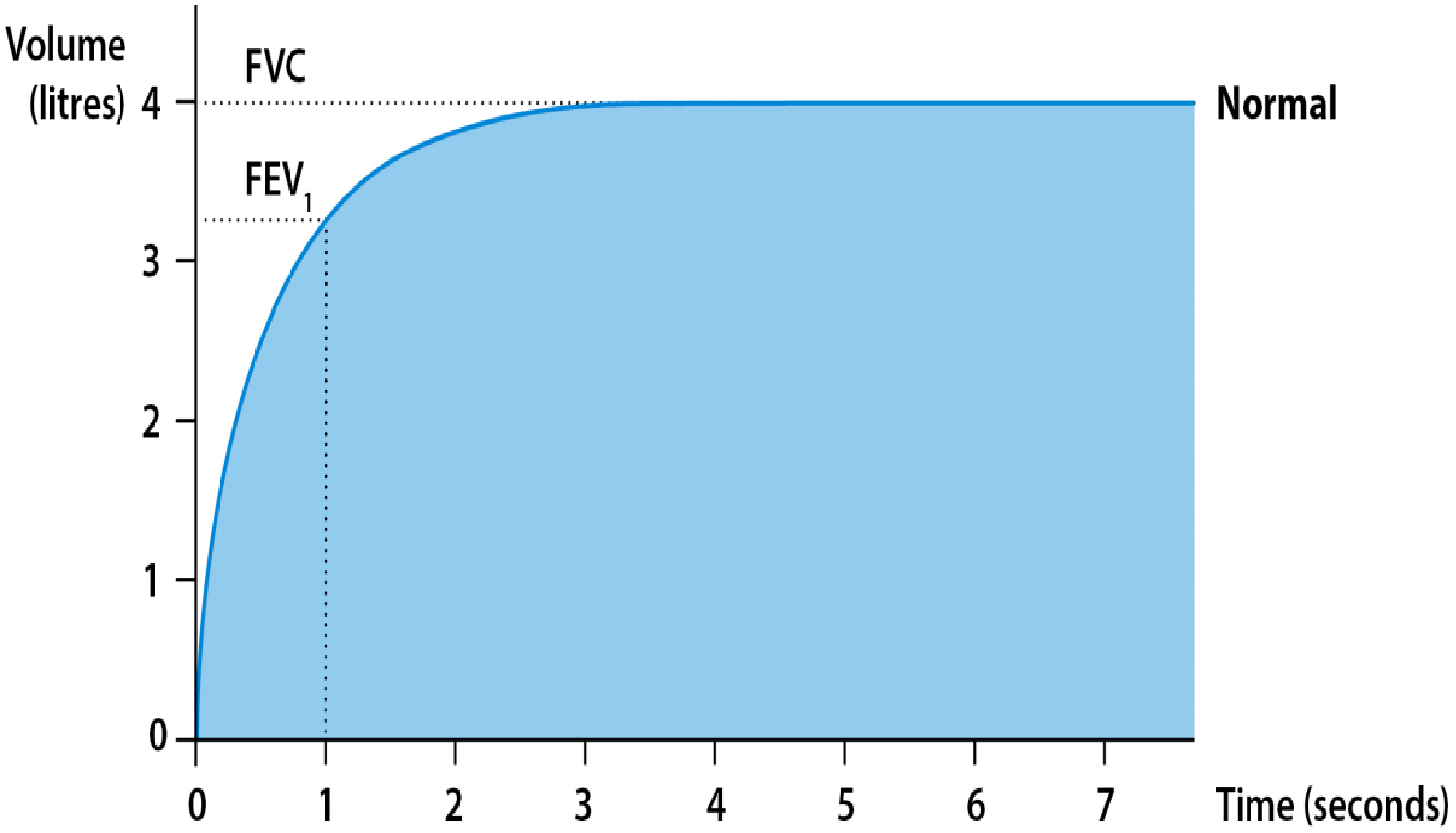

Flow-volume loop

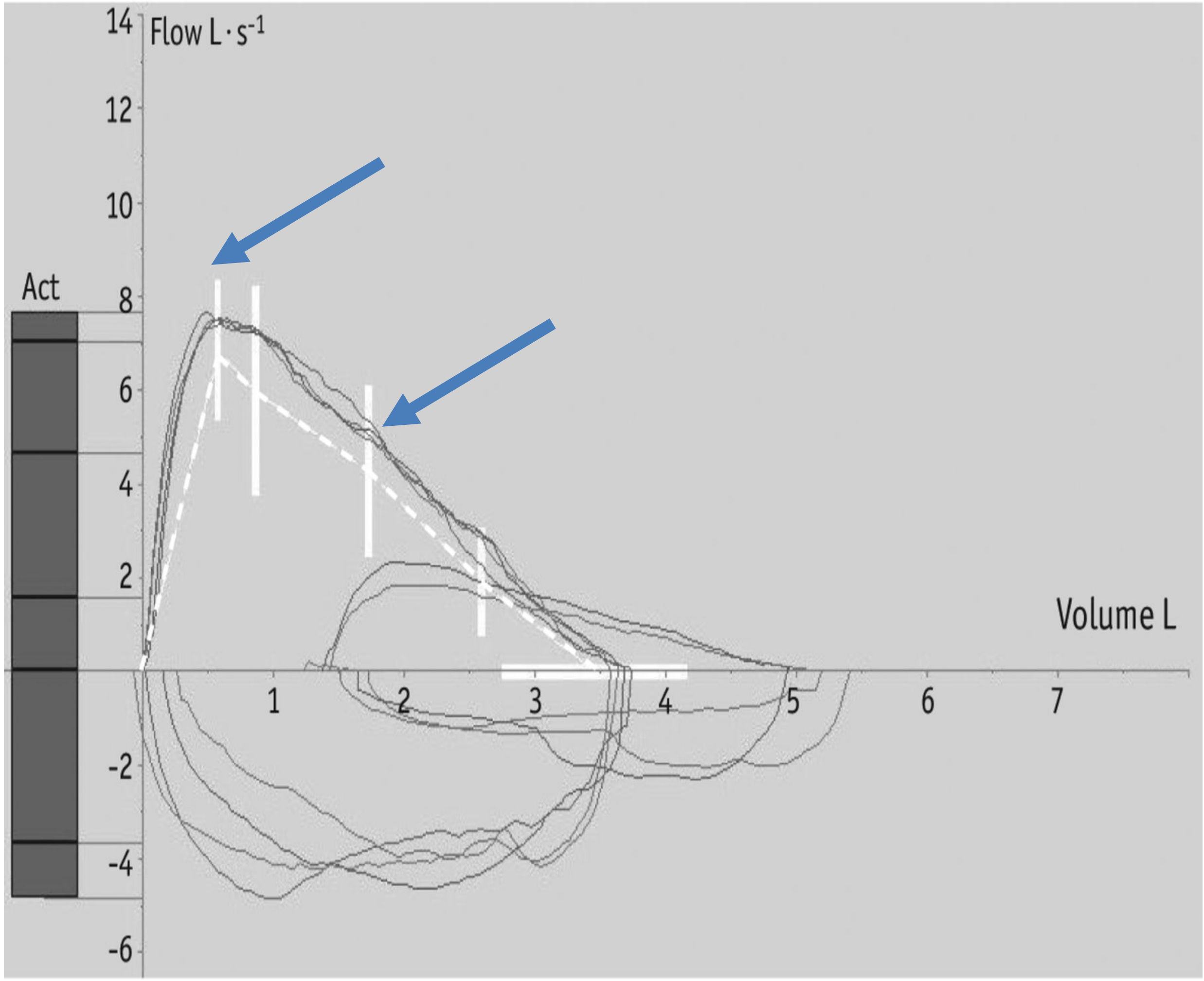

Interpretation of Tests

- Configuration of flow-volume curve
  - Look at the shape and contour of the loop
- Look at the forced expiratory time (FET)
  - Is it > 4 sec for kids and > 6 sec for adults?
- Relationship between FVC, FEV_1, $FEV_{25\text{-}75\%}$
- Response to inhaled bronchodilators

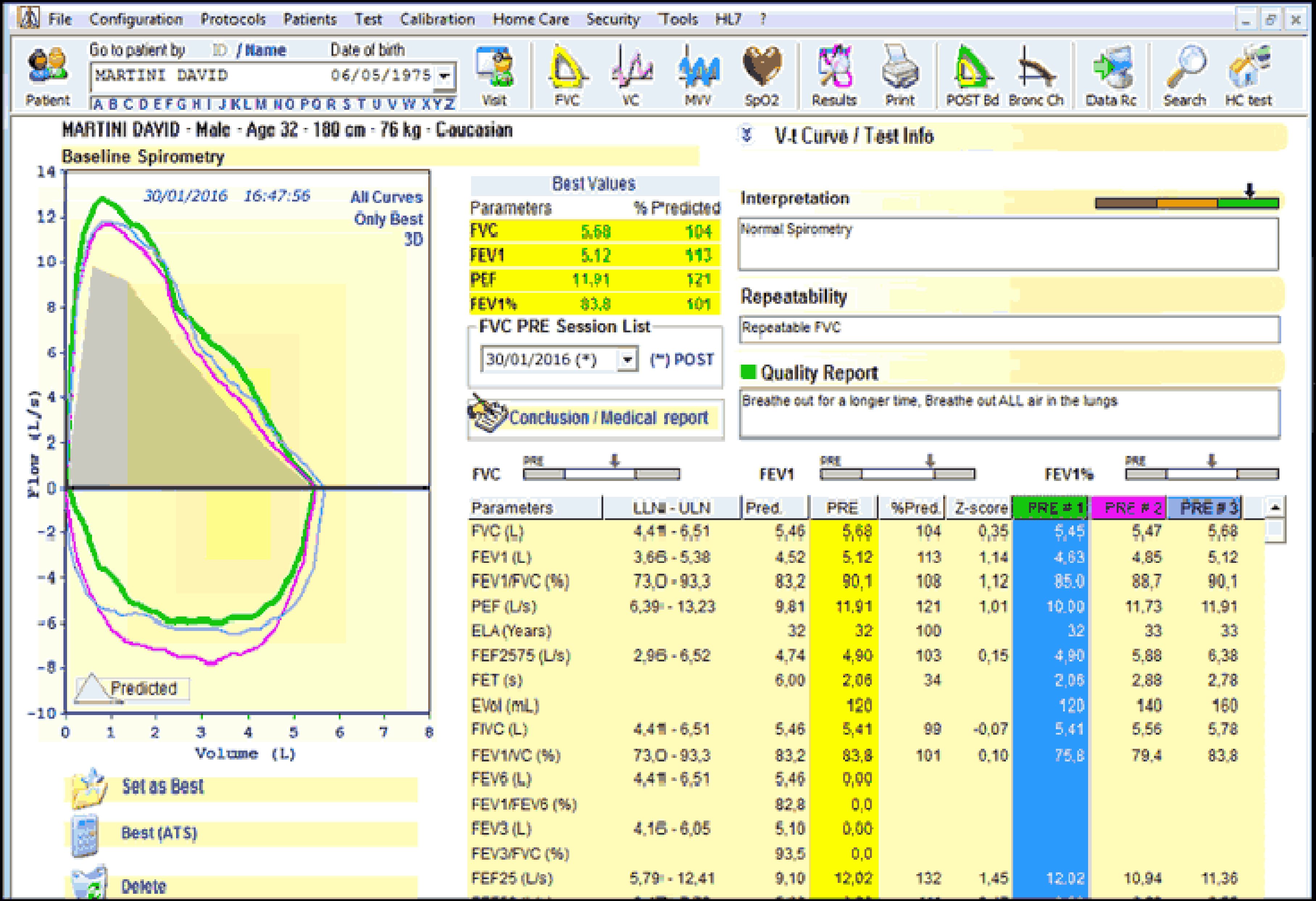
File Configuration Protocols Patients Test Calibration Home Care Security Tools HL7 ?
Go to patient by ID / Name Date of birth
MARTINI DAVID 06/05/1975
Patient Visit FVC VC MVV SpO2 Results Print POST Bd Bronc Ch Data Rc Search HC test
MARTINI DAVID - Male - Age 32 - 180 cm - 76 kg - Caucasian
Baseline Spirometry
30/01/2016 16:47:56
All Curves
Only Best
3D
Predicted
Flow (L/s)
Volume (L)
Set as Best
Best (ATS)
Delete
Best Values
Parameters % Predicted
FVC 5,68 104
FEV1 5,12 113
PEF 11,91 121
FEV1% 83,8 101
FVC PRE Session List
30/01/2016 (*) (*) POST
Conclusion / Medical report
V-t Curve / Test Info
Interpretation
Normal Spirometry
Repeatability
Repeatable FVC
Quality Report
Breathe out for a longer time, Breathe out ALL air in the lungs
FVC FEV1 FEV1%
Parameters LLN - ULN Pred. PRE %Pred. Z-score PRE # 1 PRE # 2 PRE # 3
FVC (L) 4,41 - 6,51 5,46 5,68 104 0,35 5,45 5,47 5,68
FEV1 (L) 3,66 - 5,38 4,52 5,12 113 1,14 4,63 4,85 5,12
FEV1/FVC (%) 73,0 - 93,3 83,2 90,1 108 1,12 85,0 88,7 90,1
PEF (L/s) 6,39 - 13,23 9,81 11,91 121 1,01 10,00 11,73 11,91
ELA (Years) 32 32 100 32 33 33
FEF2575 (L/s) 2,96 - 6,52 4,74 4,90 103 0,15 4,90 5,88 6,38
FET (s) 6,00 2,06 34 2,06 2,88 2,78
EVol (mL) 120 120 140 160
FIVC (L) 4,41 - 6,51 5,46 5,41 99 -0,07 5,41 5,56 5,78
FEV1/VC (%) 73,0 - 93,3 83,2 83,8 101 0,10 75,8 79,4 83,8
FEV6 (L) 4,41 - 6,51 5,46 0,00
FEV1/FEV6 (%) 82,8 0,0
FEV3 (L) 4,16 - 6,05 5,10 0,00
FEV3/FVC (%) 93,5 0,0
FEF25 (L/s) 5,79 - 12,41 9,10 12,02 132 1,45 12,02 10,94 11,36

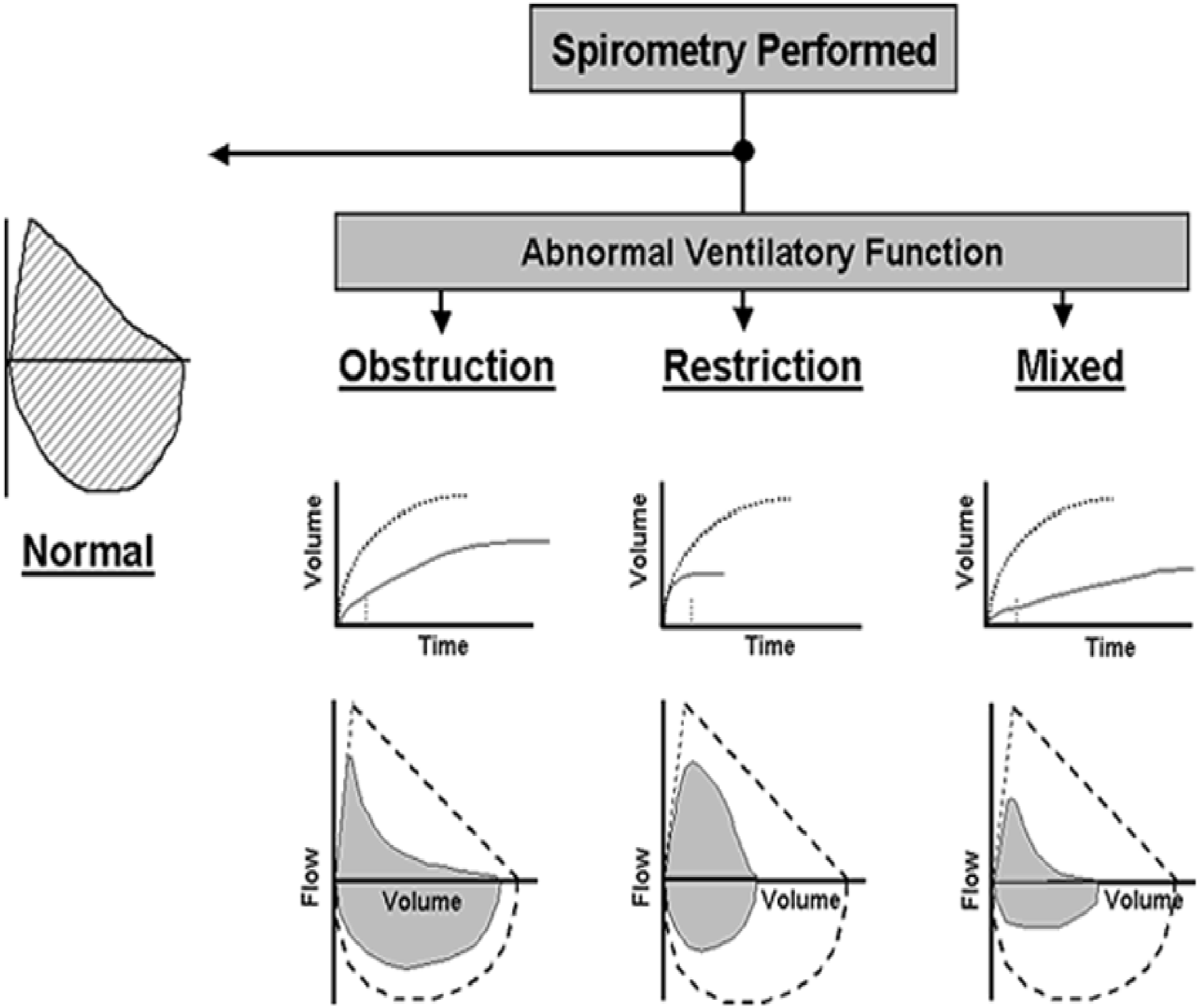
Spirometry Performed
Abnormal Ventilatory Function
Normal
Obstruction
Restriction
Mixed
Volume
Time
Volume
Time
Volume
Time
Flow
Volume
Flow
Volume
Flow
Volume

Recognition of poor quality manoeuvres

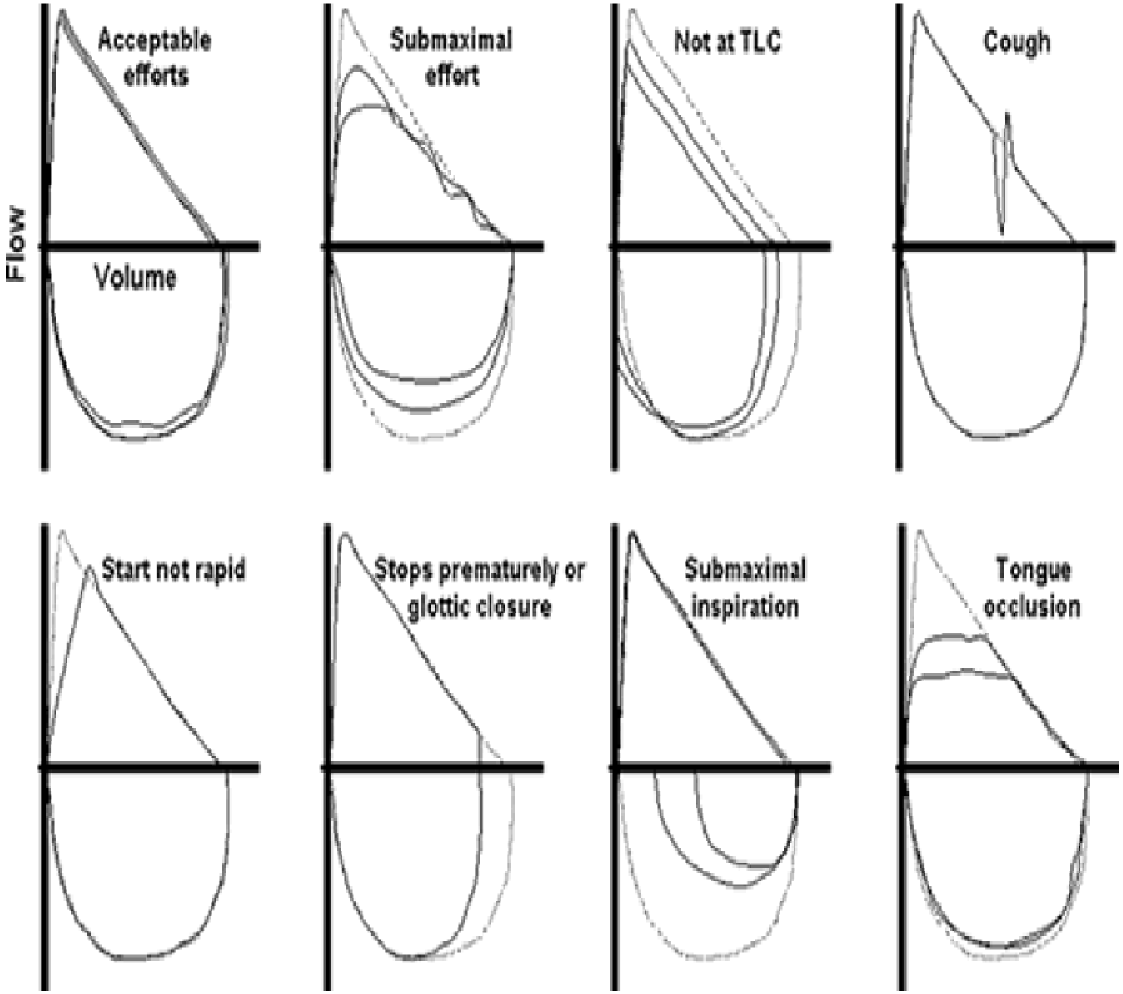

Using spirometry to comment on upper airway obstruction

Idealised examples of a) fixed, b) variable extrathoracic, and c) variable intrathoracic airway obstruction

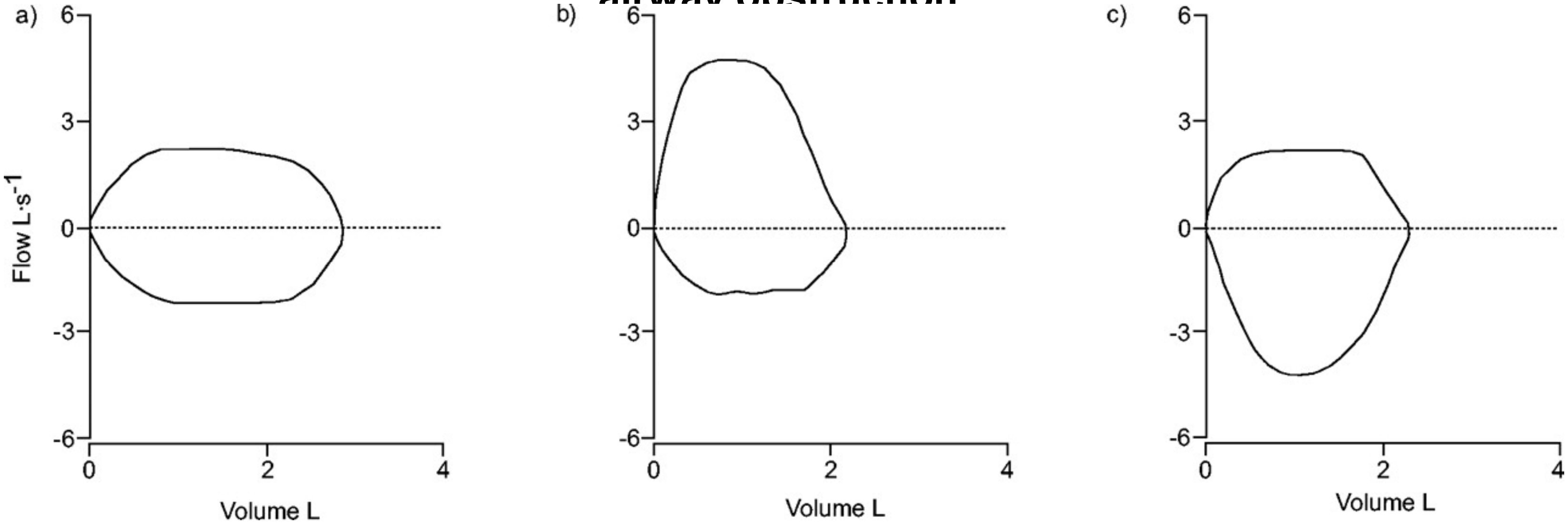

R. Pellegrino et al. Eur Respir J 2005;26:948-968

©2005 by European Respiratory Society

Unacceptable manouvers …repetition for emphasis!!!!

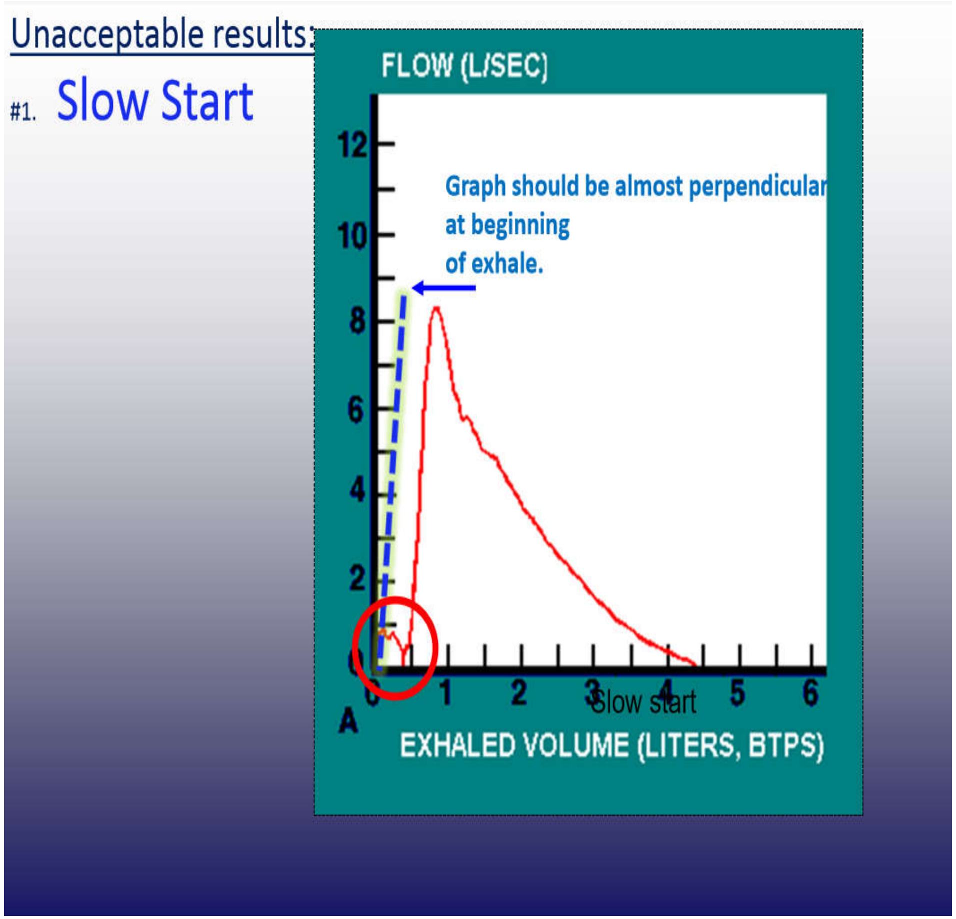

Unacceptable results:

#2. Rounded Peak

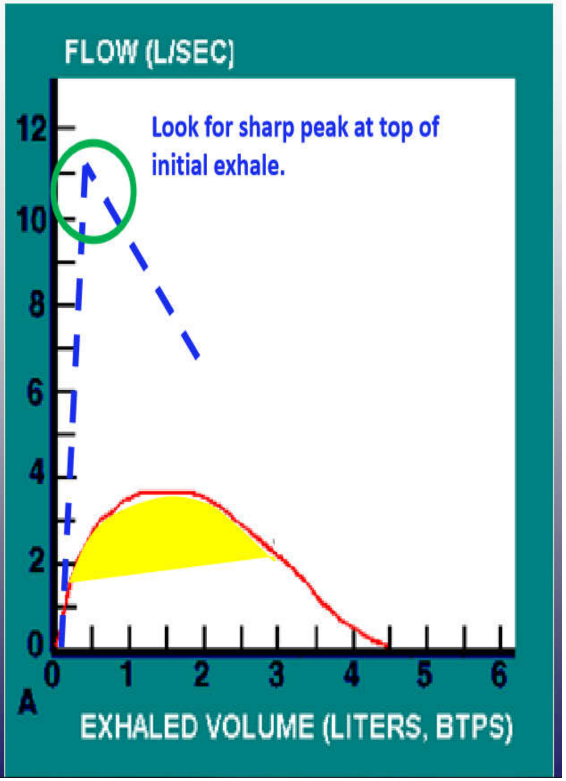

#3. Early Termination

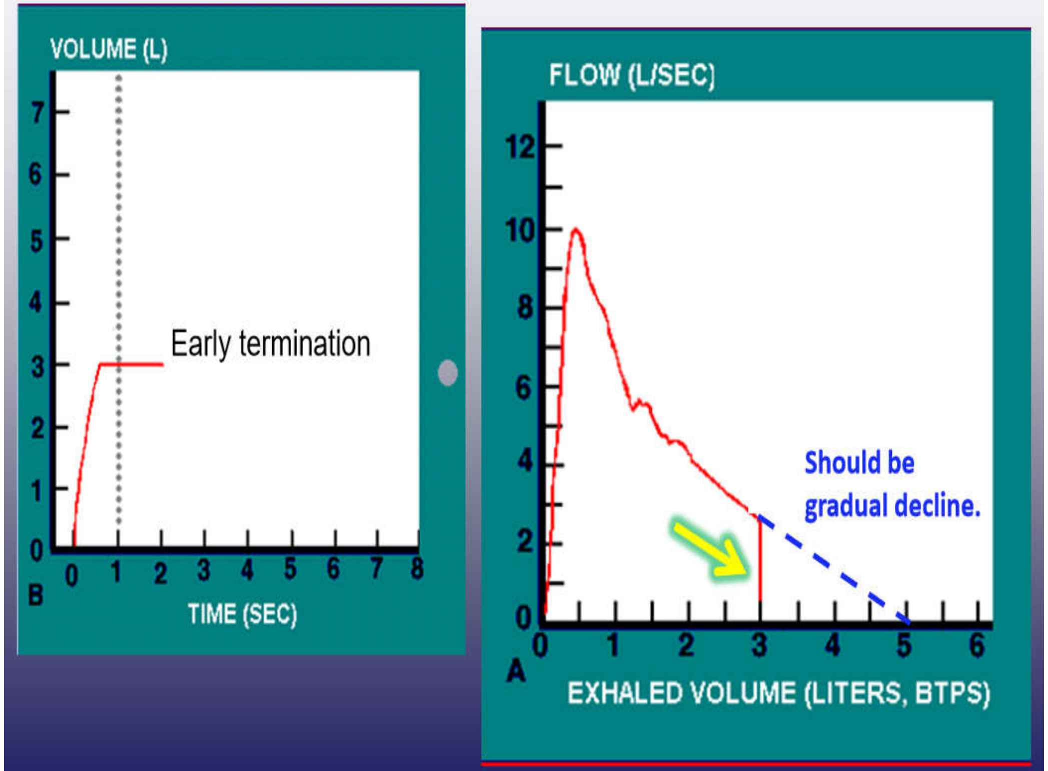

#4. # Coughing

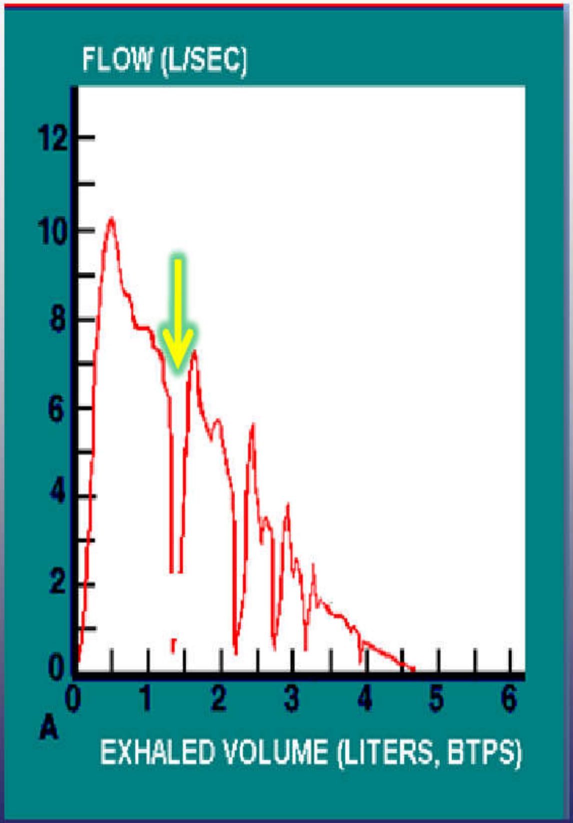

Interpreting Spirometry

- Most important factors that influence lung volumes – stature, gender, age, ethnicity
- To interpret pulmonary function tests in any individual, compare the results with reference values obtained from a well-defined population of normal subjects
  - matched for sex, age, height and ethnic origin
  - using similar test protocols;
  - and carefully calibrated/validated instruments.

A simplified algorithm that may be used to assess lung function in clinical practice.

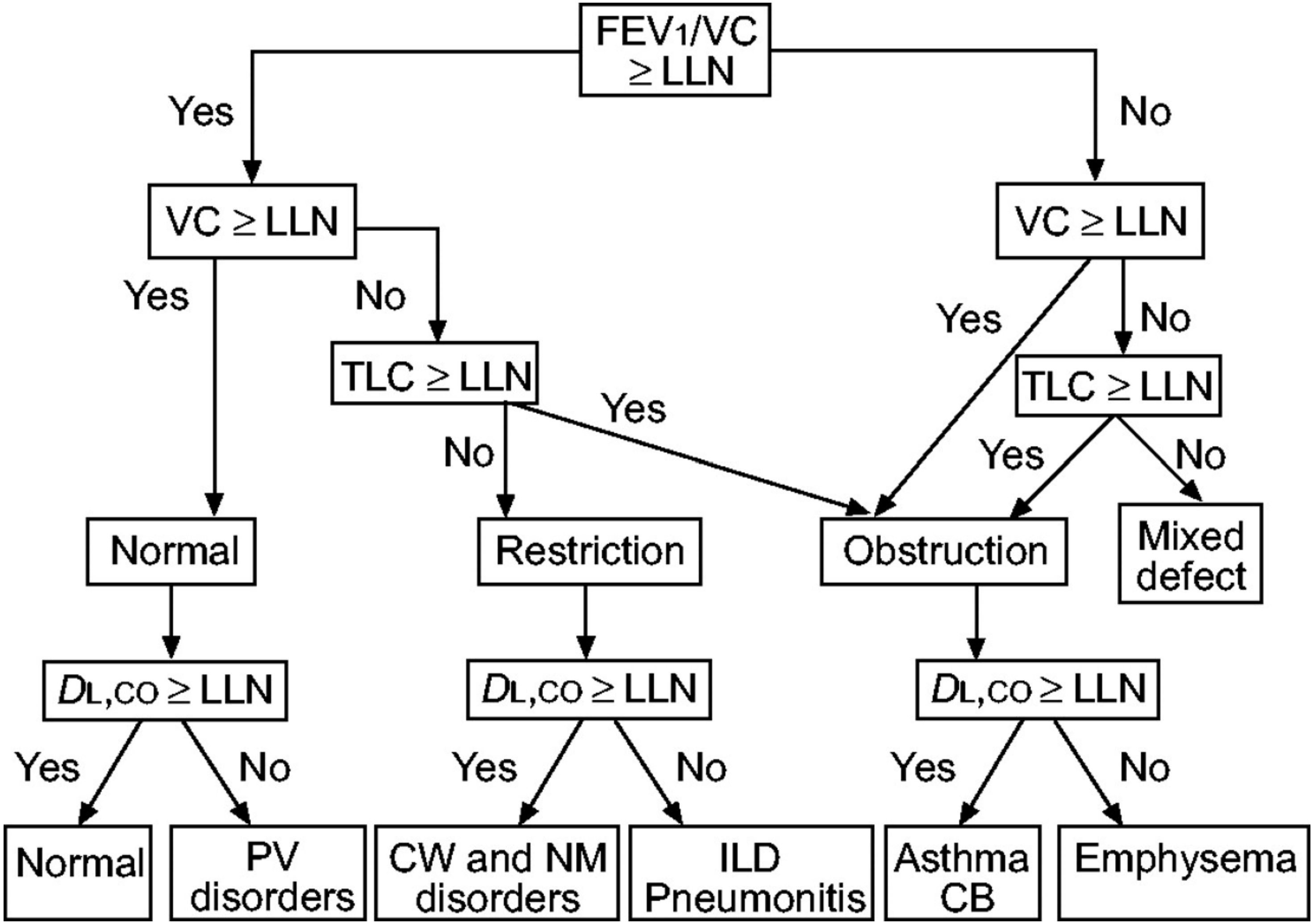

R. Pellegrino et al. Eur Respir J 2005;26:948-968

©2005 by European Respiratory Society

Reference equations

- Results are measured against a healthy population similar to the patient
  - Consider – age; height, gender and ethnicity
  - Consider the clinical history (current and past eg exprem)
- ERS/ATS/BTS • ECSC- European Coal and Steel Community •
- NHANES III • GOLD, BOLD • Tiffaneau, Knudson, Hu, Crappo, Paoletti, Polgar and Zapletal, Stanojevic
- **New GLI (Quanjer)– expressed as %predicted or Z SCORE or LLN (lower limit of normal)**

Z SCORE

FORMULA

$$z = \frac{(x - \bar{x})}{s}$$

Where:

Z = standard score/z-score

X = Raw Score

$\bar{x}$ = Mean

S = Standard Deviation

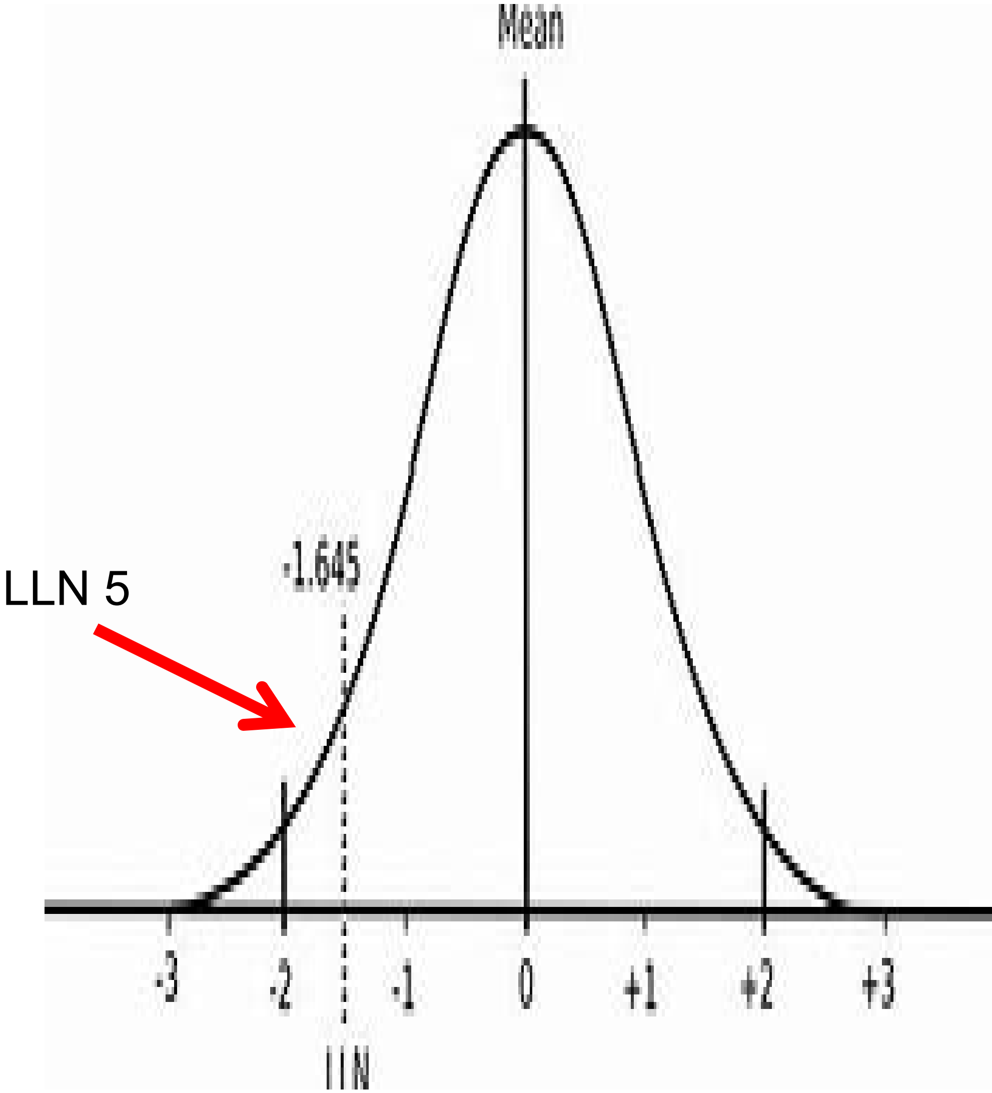

Difference between best value and best trial

- The best values (highest FVC and FEV1) obtained from **different tests** among the best acceptable trials
- The best trial/test is the test from **one acceptable** curve with the highest **SUM** of FVC and FEV1
- MOST WOULD SET THEIR MACHINES TO RECORD BEST TEST

Interpretation of the **best** test

- **Step 2.** Look at the forced vital capacity (**FVC**) to see if it is within normal limits for age, sex, race.
- **Step 1.** Look at the ratio of FEV to FVC section [remember it's the ACTUAL ratio and NOT the %predicted]
  - If FEV1/FVC is 80% (70) or higher,- it may be normal or restricted
  - If the FEV1/FVC is lower than 80 (70)% or lower, then the patient may have an obstructed lung disease (keep in mind newer LLN standard cutoffs)
- **Step 3**. Look at the forced expiratory volume in one second (**FEV1**) and determine if it is within normal limits.
- **Step 4.** If both FVC and FEV1 are normal - the patient has a normal PFT
- **Step 5.** If FVC and/or FEV1 are low, then the presence of disease is highly likely – restrictive/obstructive, mixed
  - then interpret with the ratio of FEV to FVC finding (in the absence of TLC/DLCo reading).

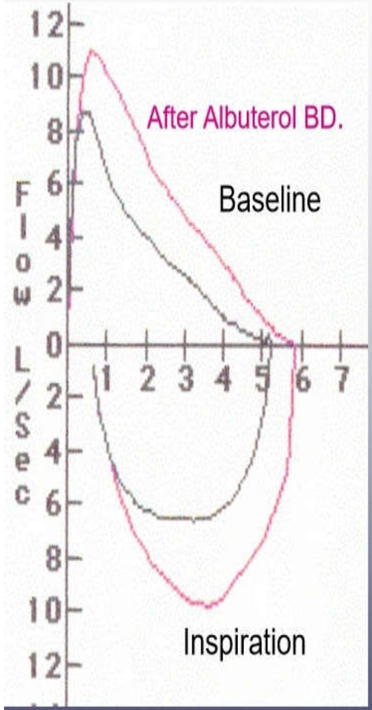

	Baseline		Post BD	
FVC	5.22	91%	5.88	103%
FEV1	3.73	78%	4.71	99%
FEV1/FVC	71%		80%	
FEFmax	8.80	101%	10.45	120%
FEF(25-75)	1.74	34%	4.18	83%

- Reversible Airflow Obstruction
- FEV1 increased 15% after bronchodilation

Quality control/quality assurance

QUALITY ASSURANCE

Quality assurance in spirometry refers to the procedures put in place in the

- ✓ environment,
- ✓ equipment,
- ✓ technician and
- ✓ the subject

before, during and after spirometry testing to reduce variability in spirometry measurements.

Quality control

- These are the checks we do to ensure that the spirometer/software is measuring accurately (truthfully) and precisely, every time
- Quality control means that regardless of any adjustment required, the process of checking that that equipment must be accomplished
- Must be applied on a **routine** basis
- It is a part of quality assurance

Calibration

- Quality assurance and calibration should not be confused
- Calibration ensures that before a spirometry test is performed the device would have been prepared to measure the volume and flow rates and they are achieved with accuracy and precision
- Prior to the actual pumping of air using the syringe a zero flow check is done by the spirometer.

- When 3 litres of air is injected (x3) into the spirometer, the spirometer should measure three litres or within + or – 3.5% (=105mls i.e 2895 – 3105mls)
  - Should measure the same results every time on every syringe

    stroke
    - This is known as precision
- For linearity testing this is done at three different flow rates
- Calibration results may suggest that an adjustment or compensation may be needed to render a spirometer as accurate and precise.
- Whilst quality control is closely tied to calibration it means more than the simple term of calibration alone.

Environmental conditions required for good outcomes in calibration/spirometry

- Ambient temperature of (17 to 35) C
- Relative humidity of (30 to 75) % RH
- Ambient pressure of (600 to 1100) hPa
- or (450 to 825) mm Hg
- or (60 to 110) kPa.
- When a spirometer is moved to a new location it is important to measure the ambient conditions

What you need for a calibration check

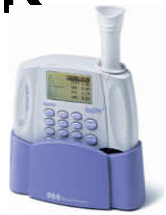

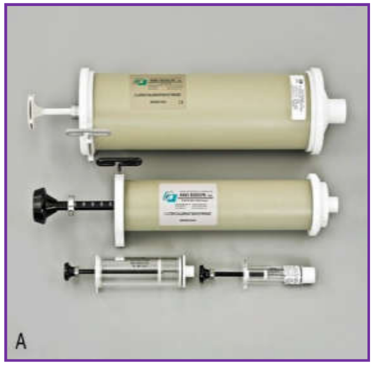

- Spirometer
- Certified 3 liter calibration syringe
- Correct adapter to syringe mouth/spirometer
- Spirometer mouthpiece
- Weather meter
  - Set spirometer on calibration Mode so that the spirometer

  does not convert the volume of dry room temperature gas

  to that at body temperature (37°C) and saturated with water vapo

  The result will be an overestimation of 4% and 9% of the volume injected

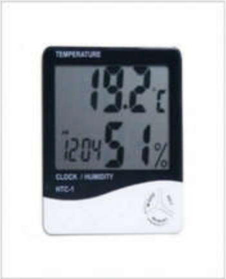

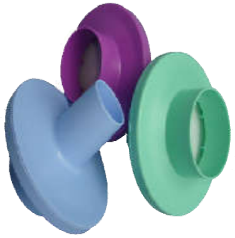

The syringe needs to be cared for

- Moving the syringe plunger in and out to check that the action is smooth, without catching or stuttering
- The syringe should be tested for leaks routinely – suggested here on a weekly basis.
  - A leak in the volume chamber will result in volume loss and inaccurate results until repairs are made.
  - The leak test is performed by placing a hand over the outlet of the syringe and pressing the syringe plunger in/out. No air should escape
- Visual inspections of the syringe on a regular basis to check for any obvious defects
- Re-validated on a yearly basis or as specified by the manufacturer.

What the calibration looks like

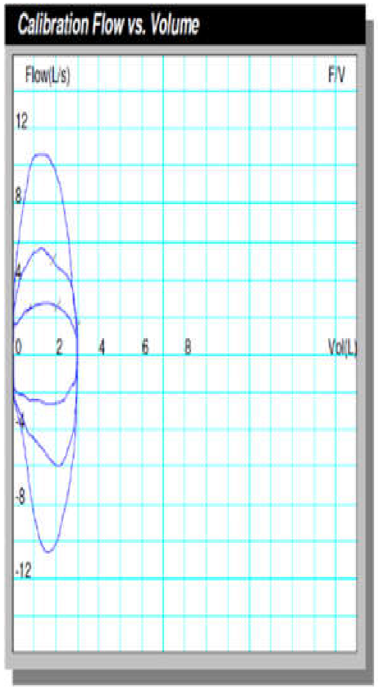

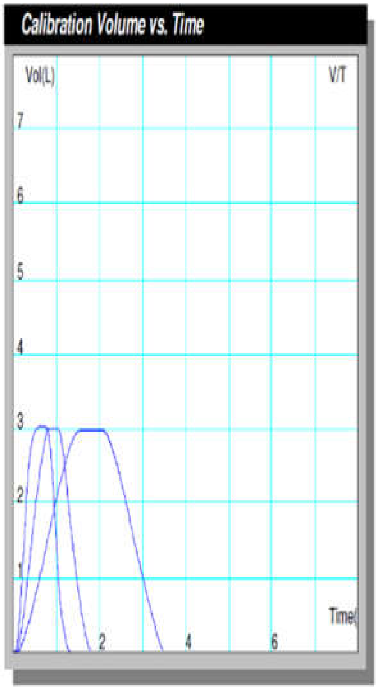

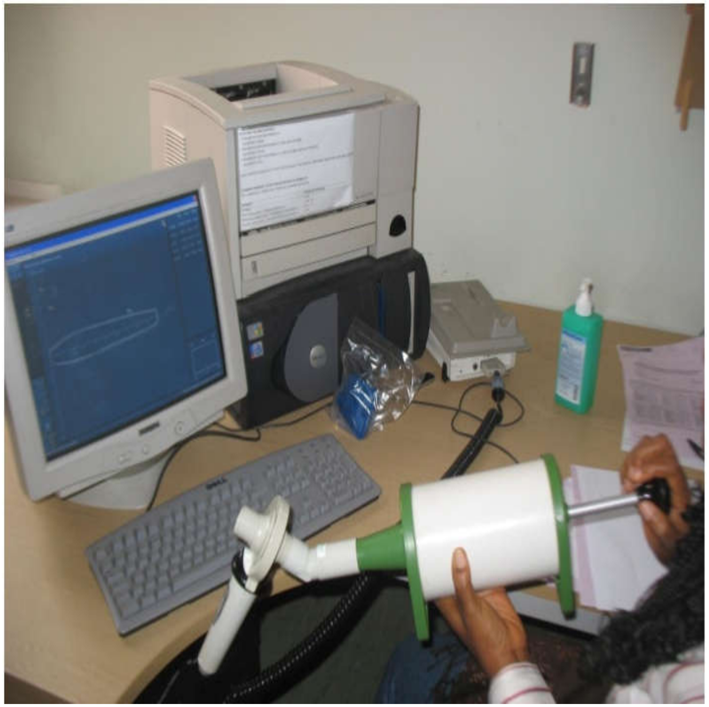

Pneumotach Calibration Report

Calibration Information

Pneumotach serial number: 2010K3716
Pneumotach ID: O-25E5-21CC-1BFC-15E6-25E5-2315-1EDF-1859-D

Pneumotach calibration date/time: 05/09/2017 02:10 PM
Pneumotach calibration expected volume (L): 3.00
Pneumotach calibration actual volume (L): 3.00

Room temperature (deg C): 22.0
Barometric pressure (mm Hg): 751.0
Relative humidity (%): 51.0

Number of efforts performed: 3
Pneumotach calibrated by: L vd Linden

Results

Result	Expd	Meas	%Expd	Meas	%Expd	Meas	%Expd
FVC (L)	3.00	3.01	100%	2.98	99%	3.00	100%
PEFR (L/s)	---	8.69	---	5.24	---	1.85	---
FEF25-75% (L/s)	---	7.73	---	4.77	---	1.64	---
FIVC (L)	3.00	2.98	99%	2.95	98%	2.95	98%

Calibration Flow vs. Volume

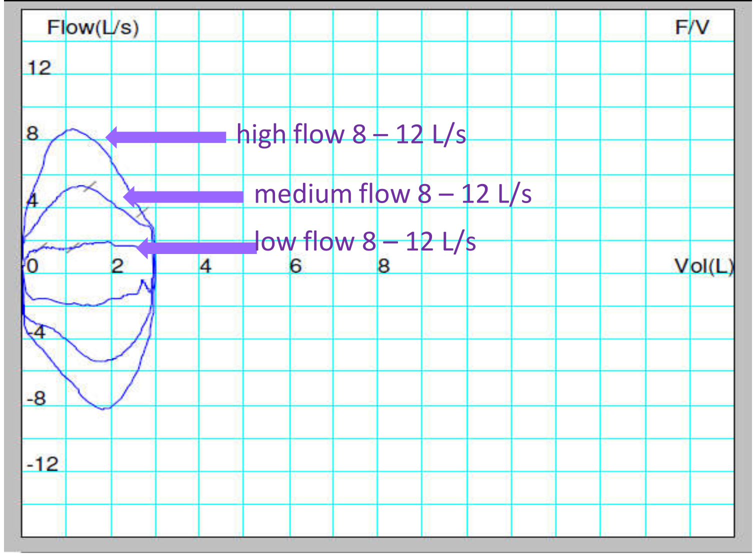

Calibration Volume vs. Time

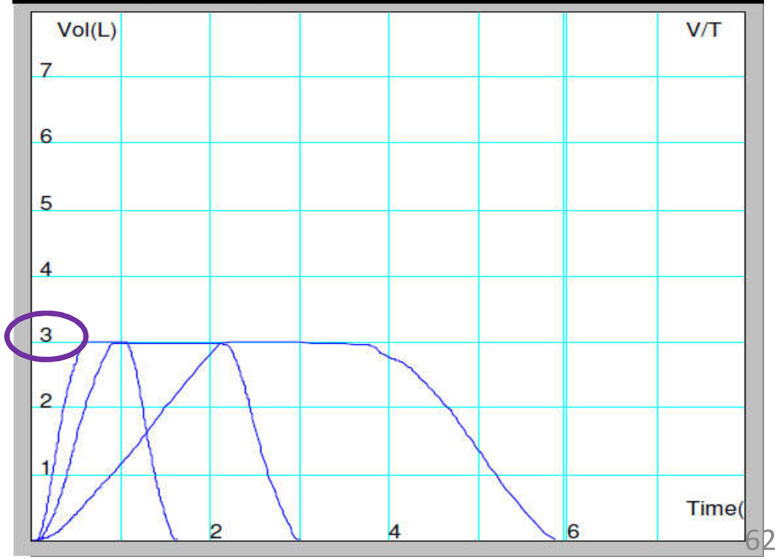

Frequency of calibration

- Depends on the location/site the spirometer is being used – hot, humid, cold, freezing
- Type of spirometer – Ultrasonic flow sensors are not affected by weather conditions in the same way a turbine or pneumotach spirometer is. The later require more robust calibration routines
- The ATS / ERS guidelines: calibration verification should take place every day in normal departments and at least twice a day in busy departments.

Biological Quality Control (BioQC)

- This involves using the lung function (instead of a calibration syringe) of a healthy non-smoking adult
  - ✓ who is readily available
  - ✓ with stable/normal lung function (PEFR above 550L/m)
  - ✓ measured/recorded on the same spirometer
  - ✓ at the same time of day
- A second BioQC subject should be identified and able to fill in if the primary BioQC subject cannot perform this function due to absence or illness

- Prior to performing spirometry on the control person one needs to determine the normal range for FVC and FEV1 for this person
  - This process known as “characterisation” is done by measuring 10 spirometry tests over a 2 week period
  - The mean value for each highest parameter is calculated by adding all 14 days of data for each value and then dividing by the number of tests done i.e. 14
  - Multiply the mean by 0.95 and 1.05 to obtain the acceptable operating range

Bronchial Dilation Studies

- Albuterol 2 puffs (MDI) use a spacer!! Test the device! or 2.5mg/ml neb –
- If there is any question that the patient got an effective dose--give another puff.
- Wait 15 mins
- Pre testing must establish a solid baseline.
- Post test needs equivalent rapid rise time and equal or greater peak flows.

Flow-volume loop

Figure 1: Example of flow-volume loop tracing. The solid trace shows FEV1 before bronchodilator administration while the dotted trace shows FEV1 10 minutes later (FEV1 increases by 18.5% from 2.7 litres to 3.3 litres). Note the slightly abnormal baseline tracing, which is concave

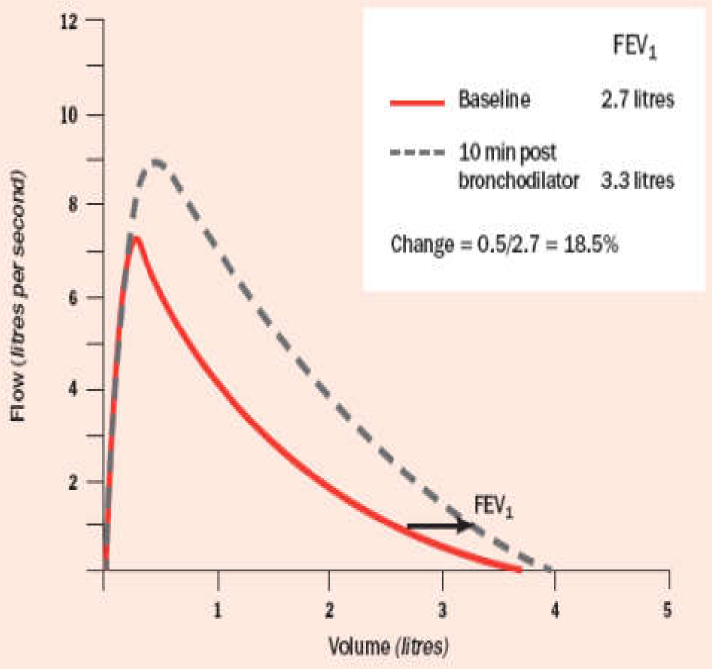

If a post bronchodilator test has been ordered, the results will show up next to the pre bronchodilator results.

	Units	Predicted	Pre Actual	Pre % Pred	Post Actual	Post % Pred.	% Change
FVC	L	5.82	4.19	72 %	3.00	52 %	-29 %
FEV1	L	4.72	1.86	39 %	2.33	49 %	26 %
FEV1/FVC	%	82 %	44 %	54 %	78 %	95 %	34 %
FEF25-75%	L/S	4.66	1.18	25 %	2.14	46 %	81 %
PEF(L/M)	L/Min	642.7	135.8	21 %	326.9	51 %	141 %
PIF	L/S						
Exp. Time	Sec.		3.60		6.18		72 %
MVV	L/Min						
VC	L	5.82					

Spirometry Criteria for Asthma

- Airflow obstruction before therapy
  - $FEV_1 < 80\%$ of predicted
  - FEV1/FVC ratio < 80%, or below lower limit of normal
- Reversibility
  - Improvement of $\geq 12\%$ in FEV_1 and/or ≥ 200 ml after treatment

Possible complications and risks

Generally safe

- SOB
- Dizziness from blow
- Trigger breathing problems
- Shakiness
- Nausea
- Fatigue

When to postpone spirometry

- Haemoptysis of unknown origin
- Pneumothorax
- Unstable cardiovascular status, recent myocardial infarction or pulmonary embolism
- Thoracic, abdominal or cerebral aneurysms
- Recent eye surgery
- Acute disorders affecting test performance, such as nausea or vomiting
- Recent thoracic or abdominal surgical procedures
- Severe chest pain
- Severely elevated blood pressure

What to consider when buying a spirometer

Feature / Specification	Explanation
Spirometer and manufacturer	Identifies each spirometer by name and gives manufacturer name
Primary Australian distributor	Name, address and contact phone number of the main supplier in Australia
Hardware	
Portable or desktop	Indicates whether or not the spirometer is designed as a portable system
Power supply	Describes the power source needed to operate the spirometer (e.g. batteries or mains power)
Sensor type	Describes the physical principle employed to measure flow/volume e.g. turbine, ultrasonic, bellows
Is external PC (not supplied) required	Indicates whether the spirometer needs a separate external computer (not supplied) to run a spirometry test
Internal or external (not suppled) printer	Indicates whether the spirometer is equipped with an internal printer or whether an external (not supplied) printer is required to print results
Weight (kg)	Total weight of spirometer in kilograms
Warranty period	Indicates time in years

What to consider when buying a spirometer

Costs	
Cost of spirometer (ex GST)	Current retail price of the spirometer in Australia as at June 2011 – GST may be payable (does not include *external* PC or *external* printer)
Cost of disposables per patient (ex GST)	Estimated retail cost of consumables per patient (e.g. mouthpieces, other disposable items)
Software	
Patient storage capacity	Indicates how many individual patient results can be stored by the spirometer
Type of database	Indicates the type of database used
Interpretation software included	Indicates if the spirometer includes software for automatic clinical interpretation of the test results
Results download to clinical software	Indicates whether the test results (numeric and/or graphic) are potentially available for download to another database (e.g. Medical Director)
Provides feedback after each blow	Indicates whether feedback is provided to encourage maximum performance during the test
Provides grading of test quality	Indicates whether the spirometer grades the overall quality of pre and post-bronchodilator trials

What to consider when buying a spirometer

Reference Values	
Reference values available	Indicates whether the operator can select from a number of normal reference studies and provides examples
Includes lower limit of normal	Indicates whether the statistically derived lower limit of normal (LLN) is printed on the final report (or used to provide the normal range) to facilitate interpretation of the measured value
Can add additional reference equations	Indicates whether the user can enter other reference equations
Can adjust for ethnicity	Indicates whether the reference values can be adjusted for ethnicity
Prints predicted flow-volume curve	Indicates whether the predicted flow-volume loop is printed on the report
General Features	
Indices measured	List of the *main* spirometric indices measured and printed/displayed by the spirometer (e.g. FEV_1, FVC, FEV_1/FVC, $FEF_{25\text{-}75\%}$, PEF)
Can user customise the final report	Indicates whether the user can customise the report (e.g. select which indices appear on the final report)
Meets 2005 ATS/ERS performance standard	Indicates whether the spirometer meets the 2005 ATS/ERS performance standards
Is printed report user definable	Indicates whether the user can customise the final printed report (e.g. user defined layout and select which indices appear)
Daily calibration check recommended	Indicates whether the manufacturer recommends that the spirometer requires its calibration to be adjusted or checked on a daily basis

What to consider when buying a spirometer

Automatically adjusts calibration	Indicates whether the accuracy of the spirometer can be adjusted by the user after calibration, or whether the adjustment can only be carried out by the distributor
Curves displayed in real-time	Indicates whether a graphic display of the blow (e.g. flow-volume and/or volume-time curve) can be viewed as the subject performs the test
Prints volume-time / flow-volume curves	Indicates whether these curves can be printed
Provides serial report	Indicates whether the results from previous tests on a patient can be incorporated into the current report
Infection control precautions	Indicates recommended method to minimise the risk of patient cross-infection (e.g. disinfection, disposable sensor, barrier filter)
Report quantifies post-BD change	Indicates whether any change in spirometry values post-bronchodilator administration can be printed on the final report to facilitate interpretation of the significance of bronchodilator response
Supplied with user manual	Indicates whether the spirometer is supplied with a user manual
Training can be provided on purchase	Indicates whether the spirometer supplier can provide basic training on the use of the spirometer on purchase

References

- ERS/ATS guidelines
- NTS guidelines

COPYRIGHT@LLA.org
